# Patterns of Intrahemispheric EEG Asymmetry in Insomnia Sufferers: An Exploratory Study

**DOI:** 10.3390/brainsci10121014

**Published:** 2020-12-19

**Authors:** Thierry Provencher, Shirley Fecteau, Célyne H. Bastien

**Affiliations:** 1 School of Psychology, Laval University, Quebec, QC G1V 0A6, Canada; Thierry.Provencher.1@ulaval.ca; 2 CERVO Brain Research Center, Centre Intégré Universitaire en Santé et Services Sociaux de la Capitale-Nationale, Quebec, QC G1V 0A6, Canada; Shirley.Fecteau@fmed.ulaval.ca; 3 Faculty of Medicine, Laval University, Quebec, QC G1V 0A6, Canada

**Keywords:** intrahemispheric asymmetry, insomnia, sleep, electroencephalography

## Abstract

Individuals with insomnia present unique patterns of electroencephalographic (EEG) asymmetry between homologous regions of each brain hemisphere, yet few studies have assessed asymmetry within the same hemisphere. Increase in intrahemispheric asymmetry during rapid eye movement (REM) sleep in good sleepers (GS) and disruption of REM sleep in insomnia sufferers (INS) both point out that this activity may be involved in the pathology of insomnia. The objective of the present exploratory study was to evaluate and quantify patterns of fronto-central, fronto-parietal, fronto-occipital, centro-parietal, centro-occipital and parieto-occipital intrahemispheric asymmetry in GS and INS, and to assess their association with sleep-wake misperception, daytime anxiety and depressive symptoms, as well as insomnia severity. This paper provides secondary analysis of standard EEG recorded in 43 INS and 19 GS for three nights in a sleep laboratory. Asymmetry measures were based on EEG power spectral analysis within 0.3–60 Hz computed between pairs of regions at frontal, central, parietal and occipital derivations. Repeated-measures ANOVAs were performed to assess group differences. Exploratory correlations were then performed on asymmetry and sleep-wake misperception, as well as self-reported daytime anxiety and depressive symptoms, and insomnia severity. INS presented increased delta and theta F3/P3 asymmetry during REM sleep compared with GS, positively associated with depressive and insomnia complaints. INS also exhibited decreased centro-occipital (C3/O1, C4/O2) and parieto-occipital (P3–O1, P4/O2) theta asymmetry during REM. These findings suggest that INS present specific patterns of intrahemispheric asymmetry, partially related to their clinical symptoms. Future studies may investigate the extent to which asymmetry is related to sleep-wake misperception or memory impairments.

## 1. Introduction

Insomnia disorder is defined primarily by a sleep-related complaint, whether it is a difficulty to initiate or maintain sleep, or the perception of the absence of restful sleep [1]. Years of research have been dedicated to pinpoint specific day- and nighttime differences between good sleepers (GS) and individuals with insomnia (INS). High-frequency activity (beta) and less slow-wave activity (delta and theta) as measured by electroencephalography (EEG) have been found to be increased in INS compared to GS [2,3,4,5,6], specifically high-frequency (beta/gamma) activity at/around sleep onset and during non-rapid eye movement (NREM) sleep [5]. However, other findings show that lower delta and greater beta activity during NREM was found only in individuals with subjective insomnia (i.e., relatively long total sleep time and relative underestimation of sleep time compared with PSG) but not objective insomnia (i.e., relatively short PSG total sleep time) [7]. Furthermore, in addition to an increase in beta power, women with insomnia, but not men, displayed more low frequency activity (delta/theta) than GS [8]. Altogether, these results suggest higher cortical arousal, especially in the beta band in INS than in GS, at sleep-onset, during sleep as well as during wake [9]. This same activity has been negatively correlated with sleep perception [4]. More recently, research has showed that compared to GS, INS present peculiarities in their rapid-eye movement (REM) sleep, known as REM sleep instability [10]. The fragmentation of this sleep stage, among others, may interfere with basic processes such as emotional regulation, which could significantly increase the risk for an INS individual to exhibit depressive symptoms [10,11]. Because anxiety, depression, and insomnia have been found to be intertwined over time, we can hypothesize that similar to depressive symptoms, anxious symptoms may also increase with REM sleep fragmentation [12]. While we may not have identified a constant pattern of abnormal EEG activity during the night yet, we know that the whole architecture of sleep is impaired in INS and may possibly play a role in sleep perception or anxiety and depression symptoms.

Mood symptoms (i.e., depression, anxiety, etc.) have been the object of many EEG studies over the years. As it is often the case, prior findings regarding EEG correlates of mood symptoms are quite heterogeneous. Several hallmark papers report the influence of brain activity on symptomatology in various mood disorders, such as generalized anxiety disorder [13], bipolar disorder [14], or major depressive disorder [15]. A significant area of research has investigated the controversial frontal alpha asymmetry hypothesis in major depressive disorder [16,17,18,19,20,21]. Results suggest that both depressive and anxiety symptomatology show moderately strong relations with right-sided frontal EEG asymmetry [21,22]. Whilst asymmetry is usually defined as the difference in brain activity between two regions, these results indicate that higher alpha activity is measured in the right hemisphere compared to the left one. Furthermore, asymmetry in alpha power originating from the parietal lobe may also play a role in depressive or anxiety symptoms [23,24,25]. In comorbid depressive and anxiety disorders, anxiety has been found to moderate the relationship between depression and frontal EEG asymmetry [26]. These findings suggest that brain activity/asymmetry may be intertwined with mood symptomatology, or at least its severity. Because depressive and anxiety symptoms are known to be highly comorbid with insomnia [27], the study of asymmetry is of particular relevance, especially since little is known regarding potential asymmetry disturbances in insomnia [28].

As indicated previously, cerebral asymmetry is operationalized as the inter- or intra-hemispheric difference in brain activity between two regions [29]. Indeed, asymmetry can be computed on homologous regions of each hemisphere (interhemispheric asymmetry, INTER). Regions of the same hemisphere can also be compared (intrahemispheric asymmetry, INTRA) [30]. To our knowledge, most INTER asymmetry studies have looked at the sleep of GS [30,31,32], and few have investigated INS sleep in this way [28,33]. What we know so far is that INTER asymmetry at central derivations (C3–C4) in INS is closely related to the stage of sleep and thus present high variability within the night [33]. Furthermore, different subtypes of insomnia, usually distinguished by the degree of corroboration between objective and subjective sleep (paradoxical, Para-I; psychophysiological, Psy-I), exhibit distinct patterns of asymmetry. For example, Para-I have been found to exhibit left frontal hypoactivation (F3 < F4), while Psy-I rather exhibit increased activation in their right parietal region (P4 > P3) [28]. In a sample of GS, strong positive correlations were found between waking alpha INTER asymmetry in frontal and temporal regions and alpha asymmetry in those same regions during sleep (NREM and REM), but particularly in REM sleep [31]. While waking INTRA asymmetry is also correlated to asymmetry during NREM sleep, REM sleep asymmetry is accentuated with respect to wakefulness [30]. In other words, REM sleep seems to amplify patterns of wakefulness asymmetry, at least in GS.

Although few studies investigated INTER in insomnia and found that different INS sub-types present unique patterns of cerebral asymmetry [28], none has specifically investigated INTRA in INS. In other words, we do not know yet whether INS (all sub-types confounded) present common, stable, patterns of INTRA asymmetry. Because we know that REM sleep in GS presents increased asymmetry, only for INTRA measures, and that REM is thought to be fragmented in INS, as measured by increased micro-arousals and wake periods throughout the night [10], this population may present peculiarities in terms of EEG asymmetry between two regions of the same hemisphere, which may be possibly associated with a fragmented sleep pattern. Because insomnia has high comorbidity rates with depressive/anxiety symptoms [27], which are partially correlated with EEG activity [34], and that higher score on the ISI reflect increased excitability of cortical networks [35], investigating INTRA asymmetry in INS would deepen our knowledge of EEG biomarkers in insomnia and perhaps shed some light on potential EEG-based treatment avenues (i.e., beyond cognitive behavioral therapy for insomnia, CBT-I).

Asymmetry may serve as a useful tool to detect abnormal brain activation, in order to guide further EEG-based novative treatment methods. Such treatments include neurofeedback [36] or neuromodulation techniques (transcranial direct current stimulation, transcranial alternative current stimulation, etc.) [37,38,39], and usually aim to correct abnormal differences in brain activity. Because CBT-I addressing insomnia symptoms via sleep hygiene education, behavioral components, cognitive restructuration, and other techniques is known to induce collateral changes in EEG activity, including an increase of slow wave activity and a reduction of sigma/beta activity [40], we can speculate that targeting EEG peculiarities via these novative methods may also induce changes in insomnia symptoms. Therefore, identifying specific patterns of asymmetry in INS that are associated with clinical symptoms may be a first step towards finding new targets for current and future treatments.

The objective of this current exploratory study is to evaluate and quantify intrahemispheric brain asymmetry of INS compared to that of GS in frontal, central, parietal and occipital sites to determine if they exhibit particular patterns of INTRA. Our primary hypothesis, strictly limited to fronto-parietal derivations, is that INS will have higher fronto-parietal INTRA during sleep compared to GS, because frontal and parietal regions have been found to exhibit distinct patterns of INTER asymmetry in different subtypes of insomnia [28] and that many differences in EEG activity can be found in frontal derivations compared to GS [2,3,4,5,6,7]. Furthermore, we believe that the hypothesized increase in fronto-parietal asymmetry will occur especially during REM sleep, because other findings support the claim that INTRA asymmetry is accentuated in REM with respect to wakefulness [30], and that REM sleep is believed to be fragmented in INS [10]. Because discrepancies between sleep diaries’ subjective ratings and PSG objective data are often reported in INS, including overestimation (subjective > objective) of their sleep onset latency (SOL) and wake after sleep onset (WASO) and underestimation (objective > subjective) of their total sleep time (TST) [28,29,30], we predict that intrahemispheric asymmetry measures that differ between groups (no hypothesis as to which regions) will be negatively correlated to TST and positively correlated to SOL and WASO. In other words, we think that higher asymmetry during sleep (only in INS, not GS) may result in increases in objective-subjective discrepancy. Finally, it is hypothesized that the same asymmetry measures will be positively associated with anxiety and depressive symptoms and the severity of their insomnia. Although our primary hypotheses are mainly related to fronto-parietal asymmetry, we have retained other derivations (i.e., fronto-central, fronto-occipital, centro-parietal, centro-occipital and parieto-occipital) in our analyses for exploratory purposes.

## 2. Materials and Methods

### 2.1. Study Design

Our study is nested in a cross-sectional study [28,41] that occurred in the sleep laboratory at the CERVO Brain Research Center, Division of Sleep and Evoked Potentials (Quebec, QC, Canada). We performed secondary data analysis of participants who have been recruited through local media advertisements between 2004 and 2011. The total sample included 21 GS, 26 Psy-I and 20 Para-I. The parent study [28,41] aimed to document frontal, central, and parietal (INTER) asymmetry in Psy-I and Para-I as well as controls (GS), and to compare their patterns of asymmetry to others already found in anxiety and depression. The complete protocol was approved by the Research Ethics Board of the CERVO Brain Research Center (neuroscience and mental health division). All participants gave their informed, written consent. For their participation in the study, each participant received a monetary compensation of approximately $250.

### 2.2. Participants

Three groups of participants aged between 25 and 55 years old were recruited as part of the parent study [28,41]. We excluded 5 left-handed participants (3 Para-I and 2 GS) to keep only right-handed individuals for this study. Our total sample includes 19 GS (9 males, 10 females), 26 Psy-I and 17 Para-I. Since our paper was interested in individuals with insomnia as a group, regardless of their subtype, we merged Psy-I and Para-I into one group of 43 INS (16 males, 27 females), which accounts for the difference in group sample sizes. GS had to report being generally satisfied with their sleep and thus reported no difficulty of sleep and daytime functioning (Insomnia Severity Index, ISI < 8). INS had to report sleep difficulties at least 3 times per week, for more than 3 months, and met the diagnosis criteria for (persistent) Insomnia Disorder based on the DSM-5 (Diagnostic and Statistical Manual of Mental Disorders, 5th edition) [1]. Participants were excluded if they met the diagnostic criteria for any medical, psychiatric, sleep, or neurologic conditions. The presence of other sleep disorders was assessed during a screening night (i.e., first night of our research protocol). All participants remained under the clinical threshold for any psychiatric or sleep disorders other than insomnia based on self-reported questionnaires and clinical interviews. In addition, the use of: (a) prescribed or over the counter sleep aids (in the INS group, a 2-week withdrawal was necessary before completing any questionnaire); (b) medication affecting sleep (e.g., beta-blockers, anxiolytics); or (c) alcohol to promote sleep excluded the participant. We chose two weeks as a withdrawal period based on a study with chronic benzodiazepine users where a progressive 15-day withdrawal was found to improve, by the end of the protocol, slow wave sleep, delta, and sleep quality (i.e., values were not different from those shown by control subjects) [42].

### 2.3. Research Protocol

Potential participants who met the inclusion criteria based on a brief telephone interview (i.e., age, sex, sleep complaints, medication use, etc.) received a series of mailed questionnaires to complete at home. They had to complete sleep diaries after every night for a two-week period, the Insomnia Severity Index (ISI) [43] as well as the Beck Anxiety Inventory (BAI) [44] and the Beck Depression Inventory (BDI) [45]. The following clinical interviews were then administered by a sleep specialist and a clinical psychologist: the Insomnia Diagnostic Interview (IDI) [46] and the Structured Clinical Interview for DSM Disorders (SCID-I) [47]. Therefore, a diagnosis of persistent insomnia disorder was given to participants who met the DSM-5 criteria [1], based on reported complaints of insomnia, self-reported questionnaires and clinical interviews. Participants meeting the study inclusion criteria were invited for three consecutive nights in the laboratory where polysomnography (PSG) was used. The technician installed and performed the bio-calibration of the equipment before turning off the lights. The usual sleep and wake schedule of participants as reported in the sleep diaries was accommodated as best as possible (±30 min) during laboratory nights (from 9:00 p.m.-midnight to 5:00–8:00 a.m). In other words, the laboratory sleep-wake schedule was at the most 30 min different to the habitual sleep and wake time.

### 2.4. Polysomnographic Montage

A standard PSG montage was used, including EEG (Xltek Trex HD Video Ambulatory System, Natus Neuro, Middleton, WI, USA) at the frontal (Fz, F3, F4), central (Cz, C3, C4), parietal (Pz, P3, P4), and occipital sites (O1, O2). The recordings were compared with the two reference sensors located on the two ear lobes, with serial resistances of 10 kOhms [48]. Recordings obtained after the first night were codified the next morning to exclude participants with other sleep disorders. Respiration and tibialis electromyography were monitored during the first night of PSG recording in order to rule out sleep apnea (apnea–hypopnea index >15) and periodic limb movements (myoclonic index with arousal >15). Participants diagnosed with another sleep disorder were excluded and referred to an appropriate sleep specialist. A Grass model 15A54 amplifier system was used (gain = 10,000, bandwidth = 0.3–100 Hz, Astro-Med, Inc., West Warwick, RI, USA) and the signals were digitized at a sampling rate of 512 Hz, using commercial software (Harmony, Stellate System, Montreal, QC, Canada). The filters (low-filter = 60 Hz, and high-filter = 0.3 Hz) of the bandwidth were optimally set for sleep recording by a technician. EEG recordings at central and occipital sites were visually assessed by trained technicians according to standard criteria using 20-s epochs to code for sleep stages [49]. Reliability checks regarding sleep stages were conducted by an independent scorer to insure a minimum of 90% interscorer agreement. Even if sleep scoring was done before the publication of the most recent guidelines, we chose to keep the original scoring since this one may be more appropriate when research uses quantitative analyses of the EEG or finer techniques of EEG analyses (e.g., event-related potentials; ERPs). Artifacts were automatically detected and rejected from the analyses [50]. The clinical data needed for the study were collected from the second and third nights.

### 2.5. Power Spectral Analysis (PSA)

PSA was conducted at a sampling rate of 512 Hz, using HarmAct 6.22 software (CARSM, Sacré-Coeur Hospital, Montreal, QC, Canada) on all EEG sites. Selection of portions of the nights for PSA performed by PSG technologists and included parts of each sleep stage (N1, N2, N3 and REM), excluding mini-arousals (0.1–7 s), micro-arousals (7.1–14.9 s), and arousals (15 s and longer), movement time, movements, or artifacts, as well as the 5-min before and after a stage shift. Hence, to respect these criteria, we required uninterrupted periods longer than 10 min. However, if no such uninterrupted period was available for selection in a cycle, a portion of this sleep stage was still selected although excluding the first and last 40 s. These criteria were followed to include consolidated sleep within each stage and exclude transitional stages. Reliability checks regarding PSA were conducted by an independent scorer to insure a minimum of 90% interscorer agreement. Based on EEG recording and analysis for sleep research guidelines [51], Fast Fourier transformations were performed over consecutive 4-sec windows with 2-sec overlap. The absolute power values for each 0.25-Hz bins were averaged to obtain the following frequency bands: slow waves (0.3–1 Hz), delta (1–4 Hz), theta (4–8 Hz), alpha (8–12 Hz), sigma (12–16 Hz), beta1 (14–20 Hz), beta2 (20–35 Hz), and gamma (35–60 Hz). While both absolute and relative EEG power can be generated with PSA, using either measures in a study with INS may sometimes lead to similar findings [7]. Hence, similar to the approach taken by Buysse and colleagues [8], our primary analyses included absolute EEG power. We also conducted analyses using relative EEG data for exploratory purposes. Although these results are not presented in this paper, complete data is available in Supplementary Materials. Asymmetry scores were computed between pairs of EEG sites using the following formula: Anterior_power_/Posterior_power_. For this project, 15 pairs of electrodes have been selected. The choice of these electrodes was based on several other studies in the field of asymmetry [28,52,53,54,55]. Different permutations of the frontal, central, parietal and occipital regions have been calculated in order to test our hypotheses: Fronto-central [Left (F3/C3), Right (F4/C4); Mid (Fz/Cz)], Fronto-parietal [Left (F3/P3), Right (F4/P4); Mid (Fz/Pz)], Fronto-occipital [Left (F3/O1), Right (F4/O2)], Centro-parietal [Left (C3/P3), Right (C4/P4); Mid (Cz/Pz)], Centro-occipital [Left (C3/O1), Right (C4/O2)], and Parieto-occipital [Left (P3/O1), Right (P4/O2)]. 

### 2.6. Statistical Aanalyses

#### 2.6.1. Sample Size

We based our sample size calculation on a study conducted by St-Jean and colleagues [28], in which they found that insomnia subtypes (Psy-I, Para-I) present distinct INTER patterns in parietal regions for high-frequency bands: beta1 (Cohen’s d = 0.554) and beta2 (Cohen’s d = 0.623). Using their lowest effect size to be conservative, a total sample size of 28 participants (at least 14 per group) was considered sufficient to analyze main group effects on INTRA with a power of 0.8 (Cohen’s d = 0.554; β = 0.2; α = 0.05) using repeated-measures ANOVAs. In the original study on which our secondary analysis is based on [28,41], three groups were recruited (19 GS, 26 Psy-I and 20 Para-I). While these groups were similar in terms of sample size, the merging of these INS subtypes (Psy-I and Para-I) in our study generated an imbalance in sample sizes between INS and GS. As indicated previously, based on our sample size calculation, we needed at least 14 participants per group for our analyses. Thus, our sample of 19 GS and 43 INS met this requirement. Despite the imbalance in sample sizes, we chose to include all participants in our analyses.

#### 2.6.2. Socio-Demographic Characteristics and Psychological Measures (Group Effects)

Groups were compared on their socio-demographic and psychological measures using multivariate analyses of variance (MANOVAs) for continuous variables and chi-square (χ2) tests for categorical variables.

#### 2.6.3. Sleep Measures (Group Effects)

Repeated-measures ANOVAs were performed to test Group effects over several relevant sleep measures: sleep onset latency (SOL), wake after sleep onset (WASO), total wake time (TWT), and total sleep time (TST). Because Para-I and Psy-I were merged to form one group (i.e., INS), GS and INS resulted in different sample sizes. However, the variance equality assumption for ANOVAs was still respected. Our data also met the other assumptions of ANOVAs: normality, sample independence, and continuous dependent variable. Therefore, we did not apply any type of statistical adjustment to different sample sizes. For these analyses, we considered two fixed factors [Group, two levels (GS, INS); Measure type, two levels (Objective, Subjective)]. Because preliminary analyses showed that Night 2 and Night 3 were not statistically different in terms of sleep measures, the factor Night [two levels (Night 2, Night 3)] was included in the model as a random effect. Then, repeated-measures ANOVAs were performed to test Group effects over several scores of sleep-wake misperception (MI), i.e., the extent to which an individual overestimate or underestimate their sleep, for the following variables: SOL, WASO, TWT, and TST. We also compared groups on the time (min) and proportion (%) spent in each stage of sleep: N1, N2, N3, and REM. For these analyses, the same factors were considered, except for the factor Measure type, which was removed because of its non-relevance (i.e., individuals cannot estimate the time they spent in stages N1, N2, N3 or REM). To avoid artificial inflation due to different sample sizes, we calculated unbiased effect sizes (Hedges’s g) to replace traditional effect sizes.

#### 2.6.4. Intrahemispheric Asymmetry (Group Effects)

Group differences in intrahemispheric asymmetry were tested individually for each selected pair of brain regions (*n* = 15) and frequency band (*n* = 8) with repeated-measures ANOVAs. Even if our sample sizes (GS and INS) were different, as indicated previously, we did not apply any type of statistical adjustment to different sample sizes. For these analyses, we considered two fixed factors [Group, two levels (GS, INS); Sleep stage, four levels (N1, N2, N3, REM)]. Because preliminary analyses showed that Night 2 and Night 3 were not statistically different in terms of asymmetry, the factor Night [two levels (Night 2, Night 3)] was included in the model as a random effect. Post-hoc F-tests were then performed to identify group differences. Although significance levels were originally set at 0.05, due to the high numbers of tests (2 levels of Group x 4 levels of Stage), we applied a conservative Bonferroni correction for type 1 error (α/8 = 0.00625). Thus, Bonferroni-corrected significance levels were set to ≤0.006. To avoid artificial inflation due to different sample sizes, we calculated unbiased effect sizes (Hedges’s g) to replace traditional effect sizes.

#### 2.6.5. Asymmetry and Misperception (Exploratory Correlations)

As an exploratory purpose, to investigate how sleep misperception and asymmetry measures may be associated, Pearson’s correlations were performed individually for each group (GS and INS) on asymmetry scores that differed between groups (i.e., statistically significant Group effects) and different sleep-wake misperception (MI) scores (i.e., Subjective_measure_/Objective_measure_), representing the extent to which an individual overestimate (MI > 1) or underestimate (MI < 1) their sleep or wake. MI scores were computed for the following variables: SOL, WASO, TST, and TWT.

#### 2.6.6. Asymmetry and Psychological Measures (Exploratory Correlations)

As an exploratory purpose, to investigate how psychological measures (depression, anxiety and insomnia) and asymmetry measures may be associated, Pearson’s correlations were performed individually for each group (GS and INS) on asymmetry scores that differed between groups (i.e., statistically significant Group effects) and scores on the BDI (depression), BAI (anxiety) and ISI (insomnia).

## 3. Results

### 3.1. Socio-Demographic Characteristics

Statistical analyses showed that GS and INS were similar according to their sociodemographic characteristics, F(4, 51) = 1.67, *p* = 0.17. Specifically, no statistical differences were found for the following variables: education, t(60) = 0.10, *p* = 0.92, and age, t(60) = −1.43, *p* = 0.16. Furthermore, no statistical difference was found regarding their sex, χ^2^(1, *n* = 62) = 0.57, *p* = 0.58. Table 1 presents means and SDs for each of the above and below variables.

INS had higher scores on the ISI compared to GS, indicating greater severity of insomnia symptoms in INS, t(59) = −16.33, *p* < 0.0001. Furthermore, INS expressed more depressive and anxiety symptoms than GS as reported on the BDI, t(56) = −4.09, *p* < 0. 001, and BAI, t(52) = −4.05, *p* < 0.001. 

### 3.2. Sleep Outcomes

Table 2 and Table 3 present means and SDs for subjective and objective sleep measures averaged across nights 2 and 3. Sleep continuity, summarized in Table 2, includes measures of SOL, WASO, TWT, and TST, both presented as subjective and objective measures, as well as their misperception index (subjective/objective). Sleep macrostructure, summarized in Table 3, includes time and proportion spent in N1, N2, N3 and REM. They are only presented as objective measures, since it is not logistically possible for an individual to estimate the time they spent in each stage of sleep.

#### 3.2.1. Sleep Continuity

Statistical analyses revealed significant Group effects for all sleep continuity variables, except for objective SOL, t(59) = −0.95, *p* = 0.35, and TST, t(60) = 0.74, *p* = 0.46. Overall, compared to GS, INS exhibited higher SOL (subjective), WASO (objective, subjective) and TWT (objective, subjective), as well as lower TST (subjective). Only for INS, statistically significant differences were found between subjective and objective measures. Results show that INS overestimated SOL, t(60) = 6.25, *p* < 0.0001, and TWT, t(60) = 9.17, *p* < 0.0001, while they underestimated TST, t(60) = −5.43, *p* < 0.0001. These results indicate that INS overestimated the time they spend awake, whereas they underestimated the time they spend sleeping.

#### 3.2.2. Sleep Macrostructure

No significant Group effect was found, meaning that time and proportion in N1, N2, N3 and REM was not statistically different between groups.

### 3.3. Asymmetry Measures

Cerebral asymmetry was computed at fronto-central, fronto-parietal, fronto-occipital, centro-parietal, centro-occipital and parieto-occipital sites. Asymmetry scores (Anterior_power_/Posterior_power_) have a theoretical value of [0, ∞+]. Scores of 1 reflect an even distribution of the proportion of a specific frequency band in the two compared regions, whereas asymmetry scores trending towards the lower and higher values of the interval represent a greater discrepancy between those same regions. Specifically, asymmetry scores lower than 1 indicate more activity (power) in the more posterior portion of the scalp, whereas asymmetry scores higher than 1 suggest more activity in the anterior portion of the scalp. In order to test the hypotheses of group differences, the effects of Group are presented below. Only statistically significant group differences under the Bonferroni-corrected threshold (*p* ≤ 0.006) are presented below and summarized in Table 4. Complete asymmetry tables are available in Supplementary Materials.

#### 3.3.1. Fronto-Central Regions

No significant Group effect was found in left (F3/C3), right (F4/C4) or mid (Fz/Cz) fronto-central asymmetry, for any of the frequency bands.

#### 3.3.2. Fronto-Parietal Regions 

**Figure 1:**
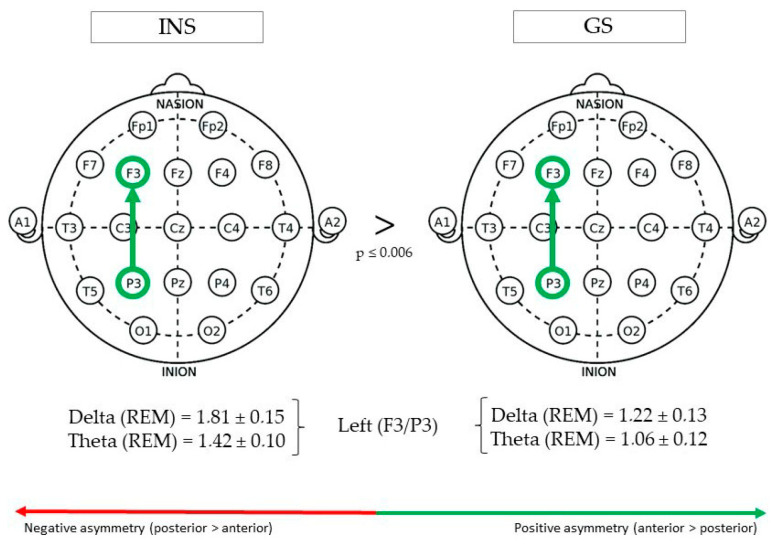
Fronto-parietal asymmetry of good sleeper and insomnia sufferers (REM/NREM).

(See Figure 1 for Significant Differences in REM/NREM Asymmetry).

During REM sleep, fronto-parietal delta asymmetry in the left hemisphere (F3/P3) was greater in INS (1.81 ± 0.15) than GS (1.22 ± 0.13), F(1,73) = 8.17, *p* = 0.005. Furthermore, theta asymmetry was also found to be greater in INS (1.42 ± 0.10) than GS (1.06 ± 0.12), F(1,62) = 7.95, *p* = 0.006. No significant Group effect was found in right (F4/P4) or mid (Fz/Pz) fronto-parietal asymmetry.

#### 3.3.3. Fronto-Occipital Regions

**Figure 2:**
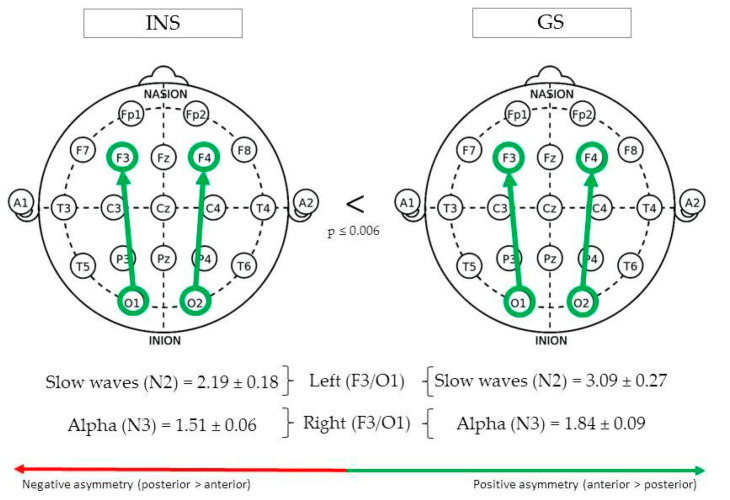
Fronto-occipital asymmetry of good sleeper and insomnia sufferers (REM/NREM).

(See Figure 2 for Significant Differences in REM/NREM Asymmetry).

During N2, fronto-occipital slow waves asymmetry in the left hemisphere (F3/O1) was greater in GS (3.09 ± 0.27) than INS (2.19 ± 0.18), F(1,135) = 7.85, *p* = 0.006. In the right hemisphere (F4/O2), absolute fronto-occipital alpha asymmetry during N3 was greater in GS (1.84 ± 0.09) than INS (1.51 ± 0.06), F(1,112) = 8.34, *p* = 0.005.

#### 3.3.4. Centro-Parietal Regions

No significant Group effect was found in left (C3/P3), right (C4/P4) or mid (Cz/Pz) centro-parietal asymmetry, for any of the frequency bands.

#### 3.3.5. Centro-Occipital Regions 

**Figure 3:**
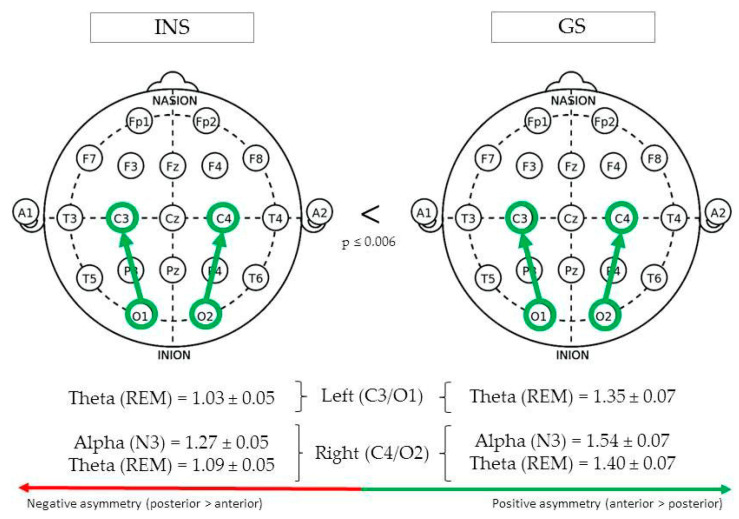
Centro-occipital asymmetry of good sleeper and insomnia sufferers (REM/NREM).

(See Figure 3 for Significant Differences in REM/NREM Asymmetry).

During REM sleep, centro-occipital theta asymmetry in the left hemisphere (C3/O1) was greater in GS (1.35 ± 0.07) than INS (1.03 ± 0.05), F(1,76) = 14.03, *p* < 0.001. In the right hemisphere (C4/O2), REM theta asymmetry was also found to be greater in GS (1.40 ± 0.07) than INS (1.09 ± 0.05), F(1,82) = 13.36, *p* < 0.001. Finally, a significant difference was found between groups for alpha asymmetry during stage N3, being greater in GS (1.54 ± 0.07) than INS (1.27 ± 0.05), F(1,96) = 9.48, *p* = 0.003.

#### 3.3.6. Parieto-Occipital Regions 

**Figure 4:**
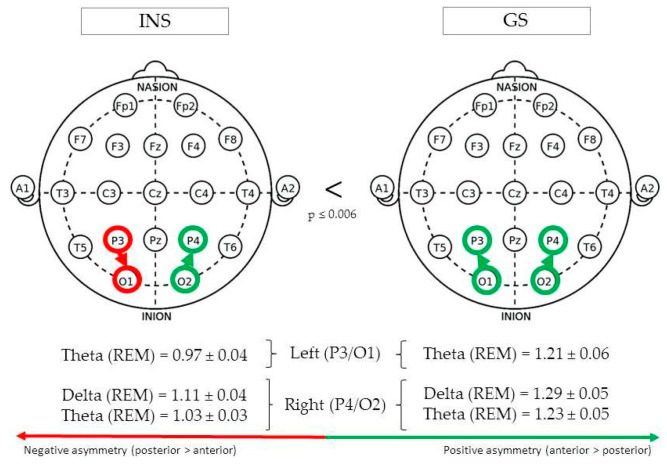
Parieto-occipital asymmetry of good sleeper and insomnia sufferers (REM/NREM).

(See Figure 4 for Significant Differences in REM/NREM Asymmetry).

During REM sleep, parieto-occipital theta asymmetry in the left hemisphere (P3/O1) was found to be greater in GS (1.21 ± 0.06) than INS (0.97 ± 0.04), F(1,73) = 12.17, *p* = 0.001. In the right hemisphere (P4/O2), REM theta asymmetry was also found to be greater in GS (1.23 ± 0.05) than INS (1.03 ± 0.03), F(1,77) = 12.41, *p* = 0.001. The same pattern was found for delta asymmetry, F(1,102) = 7.80, *p* = 0.006.

### 3.4. Correlations between Asymmetry Measures and Sleep-Wake Misperception

Asymmetry measures for which a significant Group effect has been found were retained for further analyses. We performed correlations on those measures and sleep-wake misperception outcomes (SOL, WASO, TST, TWT). A positive correlation (between 0 and 1) expresses the idea that higher asymmetry scores (i.e., increase in anterior > posterior asymmetry or decrease in posterior > anterior asymmetry) may be associated with an increase in sleep-wake misperception scores (i.e., increase in overestimation of the sleep parameter or decrease in underestimation), whereas a negative correlation (between-1 and 0) expresses the opposite idea. All significant correlation results on sleep misperception are described in Table 5.

#### 3.4.1. Sleep-Onset Latency (SOL)

SOL, often reported as “sleep latency”, represents the duration of time between when the lights are turned off (e.g., lights out) as the individual attempts to sleep, until the time they actually fall asleep, as evidenced by EEG and behavioral parameters changes consistent with sleep (e.g., sleep onset, defined as the first minute of consolidated stage 2 sleep) [56]. In GS, positive correlations between asymmetry measures and SOL misperception were found to be statistically significant. Alpha asymmetry during N3 was positively associated with SOL misperception in brain regions of the right hemisphere: F4/O2, r = 0.400, *p* < 0.05; C4/O2, r = 0.410, *p* < 0.05. Positive associations were found for theta asymmetry during REM: C3/O1, r = 0.430, *p* < 0.01; C4/O2, r = 0.343, *p* < 0.05; P3/O1, r = 0.325, *p* < 0.05; P4/O2, r = 0.401, *p* < 0.05. Finally, in INS, only delta asymmetry during REM sleep was significantly correlated with SOL misperception, although this association was, contrary to GS, negative, r = −0.259, *p* < 0.05.

#### 3.4.2. Wake after Sleep Onset (WASO)

WASO includes periods of wakefulness occurring after defined sleep onset. This parameter measures wakefulness, excluding the wakefulness occurring before sleep onset [56]. No statistically significant correlations for GS and INS were found between asymmetry measures (Fz/Cz, F3/P4, Fz/Pz, F3/O1, F4/O2, C3/O1, C4/O2, P3/O1, and P4/O2) and WASO misperception.

#### 3.4.3. Total Sleep Time (TST)

TST is the total amount of sleep time scored during the total recording time. This includes time from sleep onset to sleep offset and is distributed throughout the sleep time as minutes of N1, N2, N3, and REM sleep [56]. REM delta asymmetry was negatively associated with TST misperception in GS for left fronto-parietal regions (F3/P3), r = −0.324, *p* < 0.05, whereas a positive association was observed for INS in the right parieto-occipital regions (P4-O2), r = 0.232, *p* < 0.05.

#### 3.4.4. Total Wake Time (TWT)

TWT is the amount of wake time during the total recording time in minutes after the sleep onset and is considered as the reciprocal of total sleep time [56]. No statistically significant correlations were found between asymmetry measures and TWT misperception.

### 3.5. Correlations between Asymmetry Measures and Clinical Symptoms

The same asymmetry measures used in the previous correlation were retained for the following. We performed correlations on those measures and clinical symptoms (BAI, BDI, and ISI scores). A correlation between 0 and 1 expresses the idea that higher asymmetry scores (i.e., increase in anterior > posterior asymmetry or decrease in posterior > anterior asymmetry) may be associated with an increase in clinical symptoms, whereas a correlation between-1 and 0 expresses the opposite idea. All significant correlation results on clinical symptoms are described in Table 6.

#### 3.5.1. Beck Depression Inventory (BDI)

GS and INS presented opposite associations with asymmetry measures and BDI scores. GS exhibited statistically significant negative associations over several brain regions, whereas they were all positive in INS. In GS, negative moderate to strong associations were found with BDI scores and REM theta asymmetry (F3/P3), r = −0.559, *p* < 0.01, N2 slow waves asymmetry (F3/O1), r = −0.488, *p* < 0.01, N3 alpha asymmetry (F4/O2), r = −0.362, *p* < 0.05, as well as REM theta asymmetry (C3/O1), r = −0.331, *p* < 0.05. In INS, positive associations were found in F3/P3 (REM delta, r = 0.214, *p* < 0.05; REM theta, r = 0.205, *p* < 0.05) and P4/O2 (REM delta, r = 0.243, *p* < 0.05).

#### 3.5.2. Beck Anxiety Inventory (BAI)

We did not observe statistically significant correlations between asymmetry measures and BAI scores in GS and INS.

#### 3.5.3. Insomnia Severity Index (ISI)

Theta asymmetry during REM sleep was found to be negatively associated with ISI scores in GS, r = −0.406, *p* < 0.05, and positively in INS, r = 0.182, *p* < 0.05). In other words, higher F3 > P3 asymmetry in INS may result in higher insomnia severity. Note: although this correlation (r = 0.182) is mild compared to the other correlations aforementioned, the left fronto-parietal regions remain the only regions for which a significant correlation was found with ISI scores, both for GS and INS.

## 4. Discussion

In this study, the intrahemispheric asymmetry at frontal, central, parietal and occipital sites of good sleepers and individuals with insomnia were compared to locate potential abnormalities present at night across four stages of sleep. Also, we assessed whether or not these peculiarities in asymmetry were associated with both sleep-wake misperception and the severity of main clinical symptoms observed in insomnia. This approach was chosen to locate possible activation abnormalities in insomnia in order to identify candidate regions for potential EEG-based treatments. Such treatments may include neurofeedback, a method that assists patients to control their brain waves consciously [57], as well as non-invasive brain stimulation, a set of techniques using electric current on the surface of the head to modulate cortical excitability [58].

### 4.1. Summary of Main Findings and Comparison With Existing Literature

#### 4.1.1. Fronto-Parietal Derivations

Fronto-parietal asymmetry, specifically in the left hemisphere, showed a pattern where INS exhibited increased asymmetry in slow frequency bands (delta and theta) compared to GS, but only during REM sleep. A closer look at the results show that absolute power in their left frontal lobe was higher than in their left parietal lobe, and that this discrepancy was even higher in INS compared to GS (for which it was close to a score of 1, indicating perfect symmetry). We did not corroborate our initial hypothesis that INS would have higher fronto-parietal asymmetry during NREM sleep only, instead we found quite the opposite: this effect is only significant in REM sleep. Moreover, we found that theta asymmetry was negatively associated with BDI and ISI scores in GS, but positively in INS. Consolidating these findings show that, while slow-frequency asymmetry (frontal > parietal) is already higher in INS than GS, when theta activity favors the frontal lobe at the expense of the parietal lobe in INS, their depressive and sleep complaints are more likely to increase, whereas the opposite may happen in GS. These results seem to be partially consistent with other findings. Studies have often showed that slow and high-frequency activity are inversely correlated, meaning that while INS exhibit high beta/gamma activity, they also exhibit low delta activity [4,5]. More specifically, increased beta EEG and decreased delta activity during the sleep onset period have been observed in INS, and persisted shortly during the first NREM cycle [59]. The authors of this study even suggested that this high/low-frequency discrepancy after sleep onset may possibly play a role in the overestimation of sleep-onset latency. Because group differences in fronto-parietal asymmetry were lateralized to the left hemisphere, our results are partially in line with those of Corsi-Cabrera and colleagues [53], revealing that significantly higher levels of intrahemispheric fronto-parietal synchrony (i.e., low asymmetry) are observed in INS compared to GS in the left hemisphere only (beta and gamma bands), particularly during the wake-sleep transition.

Based on the aforementioned results, Corsi-Cabrera and colleagues [53] suggested that insomnia may present specific difficulties in deactivating frontal executive regions and in disengaging the frontal-posterior attentional network during sleep. Because these networks are thought to be involved in maintaining consciousness and internal processing [60], and that it has been previously shown that loss of consciousness is associated with decreased fronto-parietal EEG connectivity [61], an increase in slow frequency asymmetry during REM sleep can possibly influence how INS perceive their sleep. Interestingly, in a study using emotional facial expressions as stimuli, a sub-sample of individuals exhibiting both frontal alpha asymmetry and parietal asymmetry (leftward or rightward) had attentional biases toward (or away) from angry faces [62], indicating that avoidance, vigilance to threat or other attentional biases may be observed in INS. Indeed, the idea that there is an attention bias to sleep stimuli in INS has been corroborated [63,64]. In light of these findings, we may infer that such “attentional biases”, present both during the day and at night, underlie sleep-wake misperception. The persistence of this synchronously activated fronto-parietal network may underlie the mechanism involved in subjective feelings of not being asleep sometimes experienced by insomnia sufferers [53]. Interestingly, researchers have found that long-range intrahemispheric synchrony was enhanced in tonic REM states (i.e., partially preserved environmental processing) in contrast to phasic ones (i.e., functionally isolated brain state) [65]. Based on their findings, decreased intrahemispheric synchronization (i.e., greater asymmetry) may be reflected by strengthened cortical reactivity in response to external stimulation. This finding, in line with the REM sleep instability hypothesis [10], is consistent with the idea that increased fronto-parietal asymmetry exhibited by INS during REM sleep may enhance their reactivity towards their environment. The hypothesis that slow-frequency asymmetry during REM sleep may be related to sleep-wake misperception still needs to be investigated, as our study failed to establish a significant association between different sleep-wake misperception parameters (SOL, WASO, TWT, TST) and fronto-parietal asymmetry in INS. However, it is interesting to note that a negative correlation was found with GS (for delta only), indicating that the more their asymmetry increases, the more they underestimate their TST. This observation, along with the idea that increased asymmetry was found in INS, fits with the idea that increased slow-frequency fronto-parietal asymmetry in the left hemisphere is somewhat abnormal. Note that our findings on delta asymmetry may be interpreted with caution as we cannot exclude the fact that 1-4 Hz activity occurring during REM sleep may not be “pure” and may perhaps include slow potential shifts attributable to ocular artifacts [66]. Indeed, EEG contamination by ocular artifacts in the delta band is usually most pronounced in frontal leads [67]. Considering the previous results, and that slow-frequency asymmetry is also positively associated with BDI and ISI scores in INS, this pattern of increased REM sleep slow frequency asymmetry (especially theta) exhibited by INS may actually be seen as a distinctive cortical biomarker.

#### 4.1.2. Centro-Occipital and Parieto-Occipital Derivations

Every pair of regions involving occipital derivations presented left- or right-hemisphere differences between groups. It is important to note that we had no a priori hypothesis as to the direction of asymmetry in both GS and INS. Because occipital derivations are the common denominator in these pairs of brain regions, our principal finding regarding centro- and parieto-occipital asymmetry measures is that absolute theta asymmetry during REM sleep was generally higher in GS than INS (score close to 1), both in the left and right hemispheres. At left centro-occipital sites (C3/O1), only for good sleepers, theta asymmetry was positively associated with SOL misperception. Although our data does not enable us to identify why INS exhibit a decrease in theta asymmetry, our main explanation is that it may be due to an increase in theta activity in their occipital lobe and/or a decrease in theta activity in their central or parietal regions. Studies show that increases in diurnal occipital theta activity can accompany various disorders, such as mild cognitive impairment in Parkinson’s disease patients [68] or mild Alzheimer’s disease [69,70,71]. Changes in theta rhythm during REM sleep have been found to be a relevant index of cognitive impairments in the early stage of Alzheimer’s disease [72]. Knowing that episodic memory is impaired in Alzheimer’s disease, the authors suggested that an increase in theta activity may reflect a compensatory cortical mechanism to maintain cognitive processes at the early stage of brain damage. Theta activity is indeed found to positively correlate with increased workload, as well as increased fatigue [73]. These findings suggest that increased occipital theta activity in Alzheimer’s disease acts as a temporary cognitive protection factor. Because INS may exhibit a similar pattern of occipital theta activity, its role as a cognitive protection factor may be similar to that of individuals with Alzheimer’s disease. However, this remains to be validated with a sample of INS.

From another standpoint, differences between INS and GS in centro/parieto-occipital theta asymmetry during REM sleep may also be caused by a decrease in central/parietal theta activity. Researchers have found that targeted memory reactivation during sleep (i.e., cueing prior learned foreign vocabulary during sleep) increased oscillatory theta activity in central/parietal brain areas, possibly indicating an increase in memory strength and lexical integration during sleep [74,75]. Thus, a decrease in centro/parietal theta may be related to memory-weakening effects. Moreover, based on the empirically-demonstrated assumption that parietal theta is associated with memory retrieval [76,77], a neurofeedback protocol targeting theta activity in parietal regions led to improvements in memory consolidation in a classical motor task compared to a control group [78]. This neurofeedback protocol generated an initial “memory seed” that was further enhanced by sleep. In light with the above findings, we can infer that the increase in occipital theta activity during REM sleep (or the decrease in centro/parietal theta) may be in part related to deficits in memory as frequently seen in insomnia [79]. Although our study cannot confirm this hypothesis as we did not assess memory or other cognitive functions, it seems as if low centro/parietal-occipital theta asymmetry during REM sleep (as showed by our sample of INS) may be indicative of a decrease in memory consolidation, as well as an increased cognitive workload. Additional data is necessary to empirically assess this hypothesis.

### 4.2. Strengths and Limitations of the Study

Strengths. First of all, according to the most recent American Academy of Sleep Medicine (AASM) guideline [80] for the scoring of sleep and associated events, F4, C4 and O2 are the recommended EEG derivations. Thus, our choice of frontal, central, parietal and occipital derivations (left, right and mid, when available) for asymmetry measures respected these recommendations and may have helped capture a more standardized pattern of asymmetry in individuals with insomnia. Second, while many EEG studies distinguish REM and NREM sleep, we chose to sub-divide NREM into the three known stages: N1, N2 and N3. Dividing stages enabled us to better understand the complexity of EEG asymmetry. 

Limitations. As most cross-sectional studies, one of our limitations is the difficulty to establish a temporal relationship between asymmetry and insomnia or other mood symptoms. A longitudinal study, conducted with people identified as being subject to develop sleep problems, may help overcome this issue. Furthermore, although our sample meets the minimal sample size required (14 participants per group), as measured previously, we cannot exclude the fact that the magnitude of the insomnia/asymmetry association may have been slightly over-estimated. Hence, our findings should be interpreted accordingly. As highlighted by St-Jean and colleagues [28], cerebral asymmetry is a measure obtained by computing differences in cerebral activity at two electrode sites. A change in asymmetry measure can either represent an increase or a decrease in power at one site compared to the other. In our study, we used anterior/posterior asymmetry measures, but we did not directly assess if anterior activity was statistically different from posterior activity. Furthermore, while we compared anterior/posterior asymmetry between groups, we did not assess individually how frontal and parietal activity differed between them. Moreover, several repeated-measures ANOVAs were built to test our hypotheses regarding asymmetry and a conservative Bonferroni correction was applied (*p* < 0.006), meaning that we might have overlooked uncorrected group differences that were not necessarily attributable to Type I error (*p* < 0.05). Conversely, a higher number of correlation analyses were necessary to test the association between asymmetry and clinical symptoms (i.e., 60 correlations) or sleep misperception (i.e., 80 correlations), thus leading to higher risks of reporting significant results when they are not (Type I error). Because our number of correlations was high, we chose to not apply Bonferroni correction because, paradoxically, it could have led to Type II errors (i.e., fail to reject the null hypothesis H0, even if H1 is true). Because too many corrections could lead to Type II error but a lack of correction could lead to Type I error, we chose the latter, while being cautious in our interpretation of results. Therefore, results based on our correlation analyses should be seen as exploratory and be interpreted with caution. In our study, we retained only right-handed participants. Thus, our data does not enable us to assess brain laterality effects on asymmetry. For instance, our findings on left frontal/parietal asymmetry may be somewhat influenced by right-handed laterality, which could be ruled out if a similar finding would be measured in left-handed individuals. Finally, we chose to limit our study to sleep, and not wakefulness. Therefore, we cannot know whether or not certain group differences in asymmetry persist throughout the day. Future studies may be interested in assessing intrahemispheric asymmetry during the day with a sample of individuals with insomnia.

## 5. Conclusions

This study highlighted two main findings. First, insomnia presents a specific pattern of cortical asymmetry in the left fronto-parietal regions (F3/P3), where REM sleep is characterized by an increase in slow frequency (delta and theta) asymmetry. Because an association with insomnia symptoms lies within this pattern of asymmetry, it may be seen as a distinctive cortical biomarker. Second, insomnia is accompanied by lower theta asymmetry in left and right centro-occipital (C3/O1, C4/O2) and parieto-occipital (P3/O1, P4/O2) regions compared to GS. Our main explanation is that this pattern of asymmetry may play a role in memory deficits as usually seen in individuals with insomnia. These findings are quite novel as two specific patterns of intrahemispheric asymmetry were reported for INS. To our knowledge, ours is the first study to systematically assess intrahemispheric asymmetry between INS and GS. In this paper, INS presented their own pattern of asymmetry which has been associated with their sleep complaints and comorbid mood symptoms. The extent to which these asymmetry patterns are related to sleep-wake misperception or memory impairments remains to be elucidated. Future research should also formally evaluate how asymmetry exhibited by INS is associated with memory performance. More importantly, because asymmetry patterns have been linked to insomnia [28,53], as corroborated with our findings, and various mood disorders [13,14,15], future studies should investigate the etiology of these patterns. For instance, we might ask if increased fronto-parietal asymmetry occurs as part of insomnia disorder or if it is part of a prior underlying process. Although medication has not been the focus of this present study, participants being excluded if they used medication affecting sleep (e.g., anxiolytics, antidepressants), we may ask if such remedies may influence asymmetry patterns, asymmetry having been previously identified as a predictor to antidepressant treatment response and remission [81]. Thus, a future direction could be to examine the same sleep parameters as measured in our study, including EEG asymmetry, within a clinical sample of patients (insomnia depression, anxiety) who have or have never taken prescribed medication.

Based on our findings, additional studies may aim at decreasing abnormal patterns of asymmetry, so to hopefully reduce the burden of their insomnia. Because most of these techniques use at least two electrodes in their protocol (for instance, to modify cortical excitability at specific brain regions), their use in modifying EEG asymmetry is unquestionable. However, to date, few neurofeedback [57] or non-invasive brain stimulation studies [58] have targeted insomnia. According to our data, we might ask if such treatments, conjointly with CBT-I or offered as an alternative, applied on sites exhibiting cerebral asymmetry peculiarities in insomnia sufferers (e.g., left fronto-parietal, left or right centro/parieto-occipital) may reduce both their pattern of EEG asymmetry and their resulting diurnal symptoms.

## Figures and Tables

**Table 1:** Socio-demographic data of good sleepers and insomnia sufferers.

	GS (*n* = 19) Mean (SD)	INS (*n* = 43) Mean (SD)
Sex		
Male	9	16
Female	10	27
Age (years)	37.47 (9.75)	41.05 (8.79)
Education (years)	15.47 (4.01)	15.37 (3.36)
Questionnaires		
ISI	2.00 (3.04) *	16.98 (3.43)
BDI	2.11 (3.43) *	7.31 (4.99)
BAI	1.22 (2.13) *	7.33 (6.20)

GS, good sleepers; INS, individuals with insomnia; ISI, Insomnia Severity Index; BAI, Beck Anxiety Inventory; BDI, Beck Depression Inventory; * Significant group difference.

**Table 2:** Sleep continuity (mean ± SD) of good sleepers and insomnia sufferers.

	GS	INS	Group Effect *p*	Hedge’s g
SOL				
Objective	10.82 ± 3.39	14.68 ± 2.27	0.3477	1.45
Subjective	17.13 ± 5.97	35.00 ± 4.02	0.0159 *	3.81
MI (subj./obj.)	2.15 ± 0.50	5.22 ± 1.10^∆^	0.0135 *	3.20
WASO				
Objective	26.84 ± 7.91	49.98 ± 5.30	0.0182 *	3.73
Subjective	9.54 ± 9.81	59.03 ± 6.71	0.0001 *	6.37
MI (subj./obj.)	0.51 ± 0.18	2.46 ± 0.59	0.0025 *	3.87
TST				
Objective	408.33 ± 9.72	399.68 ± 6.54	0.4628	1.13
Subjective	432.51 ± 13.85	353.58 ± 9.34	<0.0001 *	7.25
MI (subj./obj.)	1.07 ± 0.03	0.89 ± 0.02^∆^	<0.0001 *	7.68
TWT				
Objective	23.53 ± 6.28	41.93 ± 4.24	0.0183 *	3.72
Subjective	33.09 ± 12.48	118.04 ± 8.51	<0.0001 *	8.61
MI (subj./obj.)	2.76 ± 0.59	6.27 ± 0.96^∆^	0.0027 *	4.05

GS, good sleepers; INS, insomnia sufferers; SOL, sleep-onset latency; WASO, wake after sleep-onset; TWT, total wake time; TST, total sleep time; * Significant difference between groups (GS vs. INS); ^∆^ Significant difference between types of measure (Subjective vs. Objective). Note: If MI (subjective/objective) > 1 = overestimation of the sleep parameter; if MI < 1 = underestimation of the sleep parameter.

**Table 3:** Sleep macrostructure (mean ± SD) of good sleepers and insomnia sufferers.

	GS	INS	Multivariate Group Effect	
			*F*	*p*
Total Time (min)				
N1	13.89 ± 2.08	12.60 ± 1.39	0.23	0.875
N2	251.91 ± 9.96	247.33 ± 6.67		
N3	38.99 ± 6.00	41.45 ± 4.01		
REM	103.68 ± 5.99	98.14 ± 4.01		
Proportion (%)				
N1	3.56 ± 0.58	3.20 ± 0.39	0.28	0.888
N2	61.72 ± 1.65	61.79 ± 1.11		
N3	9.46 ± 1.54	10.38 ± 1.03		
REM	25.26 ± 1.30	24.63 ± 0.87		

GS, good sleepers; INS, insomnia sufferers; REM, rapid eye movement sleep.

**Table 4:** Statistically significant group differences in asymmetry scores (mean ± SD) between good sleepers and insomnia sufferers under the Bonferroni-corrected threshold (*p* ≤ 0.006).

Region	Hemisphere	Stage	Frequency	GS	INS	*p*	Hedges’ g
Fronto-central	Left (F3/C3)					ns	
	Right (F4/C4)					ns	
	Mid (Fz/Cz)					ns	
Fronto-parietal	Left (F3/P3)	REM	Delta	1.22 ± 0.13	1.81 ± 0.15	0.005	4.08
		REM	Theta	1.06 ± 0.12	1.42 ± 0.10	0.006	3.38
	Right (F4/P4)					ns	
	Mid (Fz/Pz)					ns	
Fronto-occipital	Left (F3/O1)	N2	Slow waves	3.09 ± 0.27	2.19 ± 0.18	0.006	4.26
	Right (F4/O2)	N3	Alpha	1.84 ± 0.09	1.51 ± 0.06	0.005	4.69
Centro-parietal	Left (C3/P3)					ns	
	Right (C4/P4)					ns	
	Mid (Cz/Pz)					ns	
Centro-occipital	Left (C3/O1)	REM	Theta	1.35 ± 0.07	1.03 ± 0.05	<0.001	5.64
	Right (C4/O2)	N3	Alpha	1.54 ± 0.07	1.27 ± 0.05	0.003	4.76
		REM	Theta	1.40 ± 0.07	1.09 ± 0.05	<0.001	5.46
Parieto-occipital	Left (P3/O1)	REM	Theta	1.21 ± 0.06	0.97 ± 0.04	0.001	5.12
	Right (P4/O2)	REM	Delta	1.29 ± 0.05	1.11 ± 0.04	0.006	4.16
		REM	Theta	1.23 ± 0.05	1.03 ± 0.03	0.001	5.38

**Table 5:** Pearson’s correlations on intra-hemispheric asymmetry and sleep misperception across groups.

Region	Stage	Frequency	SOL		WASO		TST		TWT	
			GS	INS	GS	INS	GS	INS	GS	INS
F3/P3	REM	Delta	ns	ns	ns	ns	−0.324 *	ns	ns	ns
		Theta	ns	ns	ns	ns	ns	ns	ns	ns
F3/O1	N2	Slow	ns	ns	ns	ns	ns	ns	ns	ns
F4/O2	N3	Alpha	0.400 *	ns	ns	ns	ns	ns	ns	ns
C3/O1	REM	Theta	0.430 **	ns	ns	ns	ns	ns	ns	ns
C4/O2	N3	Alpha	0.410 *	ns	ns	ns	ns	ns	ns	ns
	REM	Theta	0.343 *	ns	ns	ns	ns	ns	ns	ns
P3/O1	REM	Theta	0.325 *	ns	ns	ns	ns	ns	ns	ns
P4/O2	REM	Delta	ns	−0.259 *	ns	ns	ns	0.232 *	ns	ns
		Theta	0.401 *	ns	ns	ns	ns	ns	ns	ns

GS = good sleepers; INS = insomnia sufferers; SOL = sleep onset latency; WASO = wake after sleep onset; TST = total sleep time; TWT = total wake time; * *p* < 0.05, ** *p* < 0.01.

**Table 6:** Pearson’s correlations on intra-hemispheric asymmetry and clinical symptoms across groups.

Region	Stage	Frequency	BDI		BAI		ISI	
			GS	INS	GS	GS	INS	GS
F3/P3	REM	Delta	ns	0.214 *	ns	ns	ns	ns
		Theta	−0.559 **	0.205 *	ns	ns	−0.406 *	0.182 *
F3/O1	N2	Slow	−0.488 **	ns	ns	ns	ns	ns
F4/O2	N3	Alpha	−0.362 *	ns	ns	ns	ns	ns
C3/O1	REM	Theta	−0.331 *	ns	ns	ns	ns	ns
C4/O2	N3	Alpha	ns	ns	ns	ns	ns	ns
	REM	Theta	ns	ns	ns	ns	ns	ns
P3/O1	REM	Theta	ns	ns	ns	ns	ns	ns
P4/O2	REM	Delta	ns	0.263 *	ns	ns	ns	ns
		Theta	ns	ns	ns	ns	ns	ns

GS = good sleepers; INS = insomnia sufferers; BDI = Beck Depression Inventory; BAI = Beck Anxiety Inventory; ISI = Insomnia Severity Index; * *p* < 0.05, ** *p* < 0.01.

## Supplementary Materials

The following are available online at https://www.mdpi.com/2076-3425/10/12/1014/s1, Table S1: Mean left fronto-central (F3/C3) asymmetry of good sleepers and insomnia sufferers; Table S2. Mean right fronto-central (F4/C4) asymmetry of good sleepers and insomnia sufferers; Table S3. Mean mid fronto-central (Fz/Cz) asymmetry of good sleepers and insomnia sufferers; Table S4. Mean left fronto-parietal (F3/P3) asymmetry of good sleepers and insomnia sufferers; Table S5. Mean right fronto-parietal (F4/P4) asymmetry of good sleepers and insomnia sufferers; Table S6. Mean mid fronto-parietal (Fz/Pz) asymmetry of good sleepers and insomnia sufferers; Table S7. Mean left fronto-occipital (F3/O1) asymmetry of good sleepers and insomnia sufferers; Table S8. Mean right fronto-occipital (F4/O2) asymmetry of good sleepers and insomnia sufferers; Table S9. Mean left centro-parietal (C3/P3) asymmetry of good sleepers and insomnia sufferers; Table S10. Mean right centro-parietal (C4/P4) asymmetry of good sleepers and insomnia sufferers; Table S11. Mean mid centro-parietal (Cz/Pz) asymmetry of good sleepers and insomnia sufferers; Table S12. Mean left centro-occipital (C3/O1) asymmetry of good sleepers and insomnia sufferers; Table S13. Mean right centro-occipital (C4/O2) asymmetry of good sleepers and insomnia sufferers; Table S14. Mean left parieto-occipital (P3/O1) asymmetry of good sleepers and insomnia sufferers; Table S15. Mean right parieto-occipital (P4/O2) asymmetry of good sleepers and insomnia sufferers.
