# Supplementary material for: Patterns of Intrahemispheric EEG Asymmetry in Insomnia Sufferers: An Exploratory Study
Source: Brain Sci. 2020 Dec 19;10(12):1014. doi: 10.3390/brainsci10121014 (PMC7766079; doi:10.3390/brainsci10121014)
Supplement: Supplementary file 1 [file brainsci-10-01014-s001.pdf]

Table S1. – Mean left fronto-central (F3/C3) asymmetry of good sleepers and insomnia sufferers.

Frequency	Stage	Type	GS		INS		*F*	Sig.
			Mean ± SD	95% CI	Mean ± SD	95% CI		
Slow waves (0-1 Hz)	N1	Absolute	1,80 ± 0,36	1,09-2,52	2,06 ± 0,25	1,57-2,55	0,35	0,554
		Relative	1,38 ± 0,08	1,23-1,54	1,44 ± 0,05	1,34-1,54	0,33	0,565
	N2	Absolute	2,41 ± 0,36	1,70-3,13	1,65 ± 0,24	1,17-2,13	3,04	0,084
		Relative	1,23 ± 0,08	1,08-1,38	1,20 ± 0,05	1,10-1,30	0,08	0,780
	N3	Absolute	1,77 ± 0,36	1,05-2,48	1,30 ± 0,25	0,80-1,79	1,15	0,285
		Relative	0,92 ± 0,08	0,77-1,07	0,95 ± 0,05	0,84-1,05	0,07	0,790
	REM	Absolute	1,73 ± 0,36	1,02-2,45	1,93 ± 0,24	1,45-2,41	0,20	0,654
		Relative	1,34 ± 0,08	1,19-1,49	1,39 ± 0,05	1,28-1,49	0,21	0,647
Delta (1-4 Hz)	N1	Absolute	1,20 ± 0,06	1,09-1,31	1,26 ± 0,04	1,19-1,34	0,79	0,374
		Relative	1,01 ± 0,03	0,95-1,07	1,03 ± 0,02	0,98-1,07	0,15	0,697
	N2	Absolute	1,38 ± 0,06	1,27-1,49	1,35 ± 0,04	1,28-1,42	0,16	0,690
		Relative	1,02 ± 0,03	0,96-1,08	1,04 ± 0,02	1,00-1,08	0,28	0,599
	N3	Absolute	1,52 ± 0,06	1,41-1,63	1,41 ± 0,04	1,34-1,49	2,55	0,113
		Relative	1,09 ± 0,03	1,03-1,15	1,08 ± 0,02	1,04-1,12	0,16	0,693
	REM	Absolute	1,21 ± 0,06	1,10-1,32	1,30 ± 0,04	1,23-1,38	1,96	0,164
		Relative	0,96 ± 0,03	0,90-1,02	0,98 ± 0,02	0,94-1,02	0,38	0,540
Theta (4-7 Hz)	N1	Absolute	1,00 ± 0,03	0,94-1,06	1,03 ± 0,02	0,98-1,07	0,52	0,474
		Relative	0,85 ± 0,03	0,78-0,92	0,85 ± 0,02	0,81-0,90	0,01	0,923
	N2	Absolute	1,01 ± 0,03	0,95-1,07	1,00 ± 0,02	0,96-1,04	0,06	0,800
		Relative	0,76 ± 0,03	0,69-0,83	0,79 ± 0,02	0,74-0,83	0,42	0,516
	N3	Absolute	1,13 ± 0,03	1,07-1,20	1,11 ± 0,02	1,07-1,15	0,46	0,501
		Relative	0,84 ± 0,03	0,77-0,91	0,88 ± 0,02	0,83-0,93	0,90	0,345
	REM	Absolute	1,07 ± 0,03	1,01-1,14	1,13 ± 0,02	1,09-1,17	2,16	0,145
		Relative	0,86 ± 0,03	0,79-0,93	0,87 ± 0,02	0,83-0,92	0,11	0,743
Alpha (7-11 Hz)	N1	Absolute	0,96 ± 0,04	0,88-1,03	0,89 ± 0,02	0,85-0,94	2,07	0,153
		Relative	0,81 ± 0,04	0,73-0,89	0,74 ± 0,03	0,69-0,80	1,85	0,175
	N2	Absolute	1,01 ± 0,04	0,94-1,08	0,98 ± 0,02	0,93-1,03	0,56	0,457
		Relative	0,75 ± 0,04	0,67-0,83	0,77 ± 0,03	0,71-0,82	0,09	0,769
	N3	Absolute	1,25 ± 0,04	1,17-1,32	1,22 ± 0,02	1,17-1,27	0,29	0,592
		Relative	0,93 ± 0,04	0,84-1,01	0,98 ± 0,03	0,93-1,04	1,36	0,246
	REM	Absolute	1,02 ± 0,04	0,95-1,09	1,01 ± 0,02	0,96-1,06	0,02	0,884

		Relative	0,81 ± 0,04	0,73-0,89	0,78 ± 0,03	0,72-0,83	0,45	0,505
Sigma (11-14 Hz)	N1	Absolute	0,97 ± 0,05	0,88-1,07	1,00 ± 0,03	0,93-1,06	0,16	0,686
		Relative	0,83 ± 0,05	0,73-0,94	0,84 ± 0,04	0,77-0,91	0,03	0,865
	N2	Absolute	1,11 ± 0,05	1,01-1,20	1,09 ± 0,03	1,02-1,15	0,15	0,704
		Relative	0,82 ± 0,05	0,71-0,92	0,85 ± 0,04	0,78-0,92	0,30	0,584
	N3	Absolute	1,22 ± 0,05	1,12-1,31	1,23 ± 0,03	1,16-1,29	0,04	0,851
		Relative	0,90 ± 0,05	0,79-1,00	0,99 ± 0,04	0,92-1,06	2,01	0,159
	REM	Absolute	0,95 ± 0,05	0,86-1,04	0,95 ± 0,03	0,89-1,01	0,00	1,000
		Relative	0,76 ± 0,05	0,66-0,86	0,74 ± 0,04	0,67-0,81	0,15	0,698
Beta1 (14-20 Hz)	N1	Absolute	0,96 ± 0,03	0,89-1,02	0,99 ± 0,02	0,95-1,03	0,79	0,378
		Relative	0,81 ± 0,03	0,75-0,88	0,83 ± 0,02	0,78-0,87	0,10	0,757
	N2	Absolute	0,75 ± 0,03	0,69-0,82	0,77 ± 0,02	0,73-0,81	0,16	0,693
		Relative	0,56 ± 0,03	0,50-0,63	0,60 ± 0,02	0,56-0,65	1,00	0,319
	N3	Absolute	0,80 ± 0,03	0,74-0,86	0,81 ± 0,02	0,77-0,86	0,11	0,742
		Relative	0,60 ± 0,03	0,53-0,67	0,66 ± 0,02	0,61-0,70	2,11	0,148
	REM	Absolute	1,08 ± 0,03	1,02-1,14	1,04 ± 0,02	1,00-1,08	1,01	0,318
		Relative	0,87 ± 0,03	0,80-0,94	0,81 ± 0,02	0,77-0,86	1,96	0,164
Beta2 (20-35 Hz)	N1	Absolute	1,11 ± 0,05	1,01-1,20	1,12 ± 0,03	1,06-1,19	0,05	0,824
		Relative	0,95 ± 0,05	0,84-1,05	0,94 ± 0,04	0,87-1,01	0,00	0,948
	N2	Absolute	1,08 ± 0,05	0,99-1,18	1,05 ± 0,03	0,99-1,12	0,22	0,640
		Relative	0,82 ± 0,05	0,71-0,92	0,83 ± 0,04	0,76-0,90	0,04	0,834
	N3	Absolute	1,06 ± 0,05	0,97-1,16	1,04 ± 0,03	0,98-1,11	0,11	0,741
		Relative	0,80 ± 0,05	0,70-0,91	0,84 ± 0,04	0,76-0,91	0,23	0,632
	REM	Absolute	1,28 ± 0,05	1,18-1,37	1,30 ± 0,03	1,24-1,37	0,16	0,686
		Relative	1,03 ± 0,05	0,92-1,13	1,02 ± 0,04	0,95-1,09	0,00	0,967
Gamma (35-60 Hz)	N1	Absolute	1,06 ± 0,05	0,96-1,15	1,11 ± 0,03	1,05-1,18	0,95	0,331
		Relative	0,90 ± 0,06	0,79-1,01	0,94 ± 0,04	0,86-1,01	0,40	0,528
	N2	Absolute	1,05 ± 0,05	0,96-1,15	1,07 ± 0,03	1,01-1,13	0,06	0,805
		Relative	0,80 ± 0,06	0,69-0,91	0,84 ± 0,04	0,77-0,92	0,51	0,476
	N3	Absolute	1,02 ± 0,05	0,93-1,12	1,11 ± 0,03	1,04-1,17	2,23	0,138
		Relative	0,78 ± 0,06	0,67-0,88	0,90 ± 0,04	0,83-0,98	3,49	0,064
	REM	Absolute	1,10 ± 0,05	1,01-1,20	1,13 ± 0,03	1,07-1,20	0,31	0,580
		Relative	0,88 ± 0,06	0,77-0,99	0,90 ± 0,04	0,82-0,97	0,07	0,789

[a] $p < 0.05$; [b] $p \leq 0.006$.

Table S2. – Mean right fronto-central (F4/C4) asymmetry of good sleepers and insomnia sufferers.

Frequency	Stage	Type	GS		INS		*F*	Sig.
			Mean ± SD	95% CI	Mean ± SD	95% CI		
Slow waves (0-1 Hz)	N1	Absolute	1,73 ± 0,23	1,28-2,19	1,78 ± 0,16	1,47-2,09	0,04	0,851
		Relative	1,40 ± 0,06	1,28-1,52	1,35 ± 0,04	1,26-1,43	0,53	0,467
	N2	Absolute	1,79 ± 0,23	1,33-2,24	1,55 ± 0,16	1,25-1,86	0,71	0,401
		Relative	1,20 ± 0,06	1,08-1,32	1,18 ± 0,04	1,10-1,26	0,07	0,787
	N3	Absolute	1,49 ± 0,23	1,04-1,94	1,24 ± 0,16	0,93-1,55	0,79	0,375
		Relative	0,94 ± 0,06	0,82-1,06	0,94 ± 0,04	0,86-1,03	0,00	0,952
	REM	Absolute	1,73 ± 0,23	1,28-2,19	1,69 ± 0,16	1,38-2,00	0,03	0,874
		Relative	1,32 ± 0,06	1,20-1,44	1,28 ± 0,04	1,19-1,36	0,31	0,579
Delta (1-4 Hz)	N1	Absolute	1,14 ± 0,04	1,06-1,21	1,20 ± 0,03	1,15-1,26	2,03	0,157
		Relative	0,99 ± 0,02	0,94-1,04	1,05 ± 0,02	1,02-1,08	4,08	**0,045**[a]
	N2	Absolute	1,30 ± 0,04	1,22-1,38	1,30 ± 0,03	1,25-1,35	0,00	0,993
		Relative	1,03 ± 0,02	0,98-1,07	1,04 ± 0,02	1,01-1,08	0,35	0,553
	N3	Absolute	1,40 ± 0,04	1,32-1,48	1,34 ± 0,03	1,29-1,39	1,60	0,208
		Relative	1,08 ± 0,02	1,03-1,13	1,06 ± 0,02	1,03-1,10	0,21	0,645
	REM	Absolute	1,23 ± 0,04	1,16-1,31	1,29 ± 0,03	1,23-1,34	1,26	0,263
		Relative	0,96 ± 0,02	0,91-1,01	1,01 ± 0,02	0,97-1,04	2,29	0,133
Theta (4-7 Hz)	N1	Absolute	0,95 ± 0,03	0,89-1,01	1,01 ± 0,02	0,97-1,05	2,57	0,112
		Relative	0,84 ± 0,05	0,74-0,94	0,89 ± 0,03	0,82-0,95	0,60	0,438
	N2	Absolute	0,97 ± 0,03	0,91-1,03	1,00 ± 0,02	0,96-1,05	0,79	0,375
		Relative	0,78 ± 0,05	0,68-0,88	0,82 ± 0,03	0,76-0,89	0,59	0,443
	N3	Absolute	1,07 ± 0,03	1,01-1,14	1,10 ± 0,02	1,06-1,14	0,42	0,520
		Relative	0,84 ± 0,05	0,74-0,94	0,89 ± 0,03	0,82-0,96	0,61	0,436
	REM	Absolute	1,04 ± 0,03	0,98-1,11	1,11 ± 0,02	1,06-1,15	2,72	0,102
		Relative	0,82 ± 0,05	0,72-0,92	0,93 ± 0,03	0,86-0,99	2,81	0,096
Alpha (7-11 Hz)	N1	Absolute	0,87 ± 0,04	0,80-0,94	0,85 ± 0,02	0,80-0,90	0,25	0,618
		Relative	0,77 ± 0,06	0,65-0,88	0,75 ± 0,04	0,67-0,83	0,07	0,790
	N2	Absolute	0,95 ± 0,04	0,88-1,02	0,95 ± 0,02	0,90-0,99	0,00	0,988
		Relative	0,76 ± 0,06	0,64-0,87	0,78 ± 0,04	0,70-0,86	0,13	0,721
	N3	Absolute	1,16 ± 0,04	1,09-1,23	1,19 ± 0,02	1,14-1,24	0,50	0,482
		Relative	0,91 ± 0,06	0,80-1,03	0,98 ± 0,04	0,90-1,06	0,78	0,377
	REM	Absolute	0,92 ± 0,04	0,85-0,99	0,93 ± 0,02	0,89-0,98	0,07	0,785
		Relative	0,73 ± 0,06	0,61-0,84	0,80 ± 0,04	0,72-0,88	1,00	0,320

Sigma (11-14 Hz)	N1	Absolute	0,89 ± 0,05	0,79-0,99	0,92 ± 0,03	0,85-0,99	0,29	0,593
		Relative	0,78 ± 0,07	0,64-0,92	0,82 ± 0,05	0,72-0,91	0,14	0,708
	N2	Absolute	1,07 ± 0,05	0,97-1,17	1,07 ± 0,03	1,01-1,14	0,00	0,969
		Relative	0,85 ± 0,07	0,71-0,99	0,88 ± 0,05	0,79-0,98	0,17	0,681
	N3	Absolute	1,15 ± 0,05	1,05-1,25	1,23 ± 0,03	1,16-1,30	1,53	0,219
		Relative	0,90 ± 0,07	0,75-1,04	1,00 ± 0,05	0,90-1,10	1,46	0,229
	REM	Absolute	0,85 ± 0,05	0,75-0,95	0,86 ± 0,03	0,79-0,93	0,01	0,909
		Relative	0,67 ± 0,07	0,53-0,81	0,75 ± 0,05	0,66-0,85	0,89	0,346
Beta1 (14-20 Hz)	N1	Absolute	0,89 ± 0,03	0,82-0,96	0,91 ± 0,02	0,87-0,96	0,31	0,581
		Relative	0,79 ± 0,08	0,63-0,94	0,81 ± 0,05	0,70-0,91	0,06	0,808
	N2	Absolute	0,75 ± 0,03	0,68-0,82	0,77 ± 0,02	0,73-0,82	0,25	0,616
		Relative	0,60 ± 0,08	0,44-0,75	0,64 ± 0,05	0,54-0,74	0,18	0,669
	N3	Absolute	0,79 ± 0,03	0,72-0,85	0,85 ± 0,02	0,81-0,90	2,60	0,110
		Relative	0,62 ± 0,08	0,47-0,77	0,70 ± 0,05	0,60-0,81	0,74	0,391
	REM	Absolute	0,98 ± 0,03	0,92-1,05	0,96 ± 0,02	0,91-1,00	0,38	0,540
		Relative	0,77 ± 0,08	0,62-0,93	0,86 ± 0,05	0,75-0,96	0,77	0,381
Beta2 (20-35 Hz)	N1	Absolute	0,99 ± 0,05	0,90-1,09	1,08 ± 0,03	1,01-1,14	1,94	0,167
		Relative	0,88 ± 0,08	0,73-1,04	0,95 ± 0,05	0,85-1,05	0,51	0,477
	N2	Absolute	0,96 ± 0,05	0,87-1,06	0,99 ± 0,03	0,93-1,06	0,20	0,654
		Relative	0,77 ± 0,08	0,62-0,93	0,82 ± 0,05	0,71-0,92	0,22	0,641
	N3	Absolute	0,95 ± 0,05	0,85-1,04	0,98 ± 0,03	0,92-1,05	0,41	0,522
		Relative	0,75 ± 0,08	0,60-0,90	0,80 ± 0,05	0,70-0,91	0,29	0,593
	REM	Absolute	1,18 ± 0,05	1,08-1,28	1,26 ± 0,03	1,19-1,32	1,73	0,191
		Relative	0,93 ± 0,08	0,78-1,08	1,08 ± 0,05	0,98-1,18	2,68	0,103
Gamma (35-60 Hz)	N1	Absolute	0,93 ± 0,04	0,86-1,00	1,03 ± 0,02	0,99-1,08	6,36	**0,013**[a]
		Relative	0,82 ± 0,04	0,74-0,90	0,92 ± 0,03	0,86-0,98	4,24	**0,041**[a]
	N2	Absolute	0,97 ± 0,04	0,90-1,04	1,00 ± 0,02	0,95-1,05	0,71	0,400
		Relative	0,78 ± 0,04	0,70-0,86	0,83 ± 0,03	0,77-0,88	0,92	0,339
	N3	Absolute	0,95 ± 0,04	0,88-1,02	0,98 ± 0,02	0,94-1,03	0,54	0,465
		Relative	0,76 ± 0,04	0,68-0,85	0,80 ± 0,03	0,75-0,86	0,59	0,443
	REM	Absolute	1,04 ± 0,04	0,97-1,11	1,05 ± 0,02	1,01-1,10	0,13	0,720
		Relative	0,82 ± 0,04	0,74-0,90	0,85 ± 0,03	0,79-0,90	0,35	0,552

[a]$p < 0.05$; [b]$p \leq 0.006$.

Table S3. – Mean mid fronto-central (Fz/Cz) asymmetry of good sleepers and insomnia sufferers.

Frequency	Stage	Type	GS		INS		*F*	Sig.
			Mean ± SD	95% CI	Mean ± SD	95% CI		
Slow waves (0-1 Hz)	N1	Absolute	1,28 ± 0,19	0,90-1,67	1,46 ± 0,13	1,20-1,72	0,59	0,445
		Relative	1,35 ± 0,06	1,23-1,48	1,41 ± 0,04	1,32-1,49	0,56	0,455
	N2	Absolute	1,53 ± 0,19	1,15-1,91	1,25 ± 0,13	0,99-1,51	1,41	0,237
		Relative	1,28 ± 0,06	1,15-1,40	1,20 ± 0,04	1,12-1,29	0,89	0,346
	N3	Absolute	1,47 ± 0,19	1,09-1,85	1,00 ± 0,13	0,73-1,26	4,08	**0,045**[a]
		Relative	0,95 ± 0,06	0,83-1,08	0,93 ± 0,04	0,85-1,02	0,07	0,794
	REM	Absolute	1,37 ± 0,19	0,98-1,75	1,44 ± 0,13	1,18-1,70	0,10	0,752
		Relative	1,36 ± 0,06	1,24-1,49	1,44 ± 0,04	1,35-1,52	0,95	0,333
Delta (1-4 Hz)	N1	Absolute	0,93 ± 0,04	0,86-1,01	0,98 ± 0,03	0,93-1,04	1,22	0,272
		Relative	1,01 ± 0,02	0,97-1,05	1,05 ± 0,01	1,02-1,08	3,13	0,079
	N2	Absolute	1,11 ± 0,04	1,03-1,18	1,07 ± 0,03	1,02-1,12	0,81	0,370
		Relative	1,02 ± 0,02	0,98-1,06	1,04 ± 0,01	1,01-1,07	0,98	0,323
	N3	Absolute	1,24 ± 0,04	1,16-1,31	1,14 ± 0,03	1,09-1,19	4,50	**0,036**[a]
		Relative	1,08 ± 0,02	1,04-1,12	1,07 ± 0,01	1,04-1,10	0,23	0,634
	REM	Absolute	0,89 ± 0,04	0,82-0,97	0,94 ± 0,03	0,89-0,99	0,98	0,323
		Relative	0,93 ± 0,02	0,89-0,97	0,95 ± 0,01	0,92-0,98	0,56	0,455
Theta (4-7 Hz)	N1	Absolute	0,76 ± 0,03	0,71-0,82	0,78 ± 0,02	0,74-0,81	0,15	0,703
		Relative	0,83 ± 0,03	0,77-0,89	0,85 ± 0,02	0,81-0,89	0,30	0,585
	N2	Absolute	0,80 ± 0,03	0,74-0,86	0,78 ± 0,02	0,75-0,82	0,28	0,601
		Relative	0,74 ± 0,03	0,68-0,80	0,77 ± 0,02	0,74-0,81	0,94	0,333
	N3	Absolute	0,90 ± 0,03	0,85-0,96	0,87 ± 0,02	0,84-0,91	0,78	0,379
		Relative	0,81 ± 0,03	0,75-0,87	0,84 ± 0,02	0,80-0,88	0,67	0,414
	REM	Absolute	0,78 ± 0,03	0,73-0,84	0,80 ± 0,02	0,77-0,84	0,32	0,575
		Relative	0,83 ± 0,03	0,77-0,89	0,82 ± 0,02	0,78-0,86	0,05	0,829
Alpha (7-11 Hz)	N1	Absolute	0,79 ± 0,03	0,73-0,85	0,74 ± 0,02	0,70-0,78	1,71	0,194
		Relative	0,87 ± 0,04	0,80-0,94	0,82 ± 0,02	0,77-0,86	1,48	0,225
	N2	Absolute	0,86 ± 0,03	0,81-0,92	0,83 ± 0,02	0,79-0,87	0,86	0,357
		Relative	0,81 ± 0,04	0,74-0,88	0,83 ± 0,02	0,78-0,87	0,18	0,668
	N3	Absolute	1,04 ± 0,03	0,98-1,09	1,00 ± 0,02	0,96-1,04	1,01	0,317
		Relative	0,93 ± 0,04	0,86-1,00	0,97 ± 0,02	0,93-1,02	0,87	0,352
	REM	Absolute	0,81 ± 0,03	0,75-0,86	0,80 ± 0,02	0,76-0,83	0,09	0,767
		Relative	0,85 ± 0,04	0,78-0,92	0,81 ± 0,02	0,77-0,86	0,62	0,433

Sigma (11-14 Hz)	N1	Absolute	0,79 ± 0,03	0,72-0,85	0,81 ± 0,02	0,77-0,86	0,38	0,539
		Relative	0,87 ± 0,04	0,80-0,94	0,89 ± 0,02	0,84-0,94	0,29	0,589
	N2	Absolute	0,86 ± 0,03	0,79-0,92	0,84 ± 0,02	0,79-0,88	0,20	0,655
		Relative	0,79 ± 0,04	0,72-0,87	0,83 ± 0,02	0,78-0,88	0,58	0,447
	N3	Absolute	0,89 ± 0,03	0,82-0,96	0,89 ± 0,02	0,85-0,94	0,01	0,931
		Relative	0,79 ± 0,04	0,72-0,87	0,86 ± 0,02	0,81-0,91	2,15	0,145
	REM	Absolute	0,76 ± 0,03	0,69-0,82	0,77 ± 0,02	0,72-0,81	0,06	0,801
		Relative	0,80 ± 0,04	0,73-0,87	0,78 ± 0,02	0,74-0,83	0,11	0,742
Beta1 (14-20 Hz)	N1	Absolute	0,72 ± 0,02	0,67-0,77	0,74 ± 0,02	0,70-0,77	0,19	0,664
		Relative	0,80 ± 0,03	0,74-0,85	0,81 ± 0,02	0,77-0,85	0,11	0,744
	N2	Absolute	0,57 ± 0,02	0,52-0,62	0,58 ± 0,02	0,55-0,62	0,30	0,584
		Relative	0,52 ± 0,03	0,47-0,58	0,58 ± 0,02	0,54-0,62	2,29	0,132
	N3	Absolute	0,58 ± 0,02	0,53-0,63	0,60 ± 0,02	0,57-0,63	0,55	0,461
		Relative	0,52 ± 0,03	0,46-0,58	0,58 ± 0,02	0,54-0,62	2,99	0,086
	REM	Absolute	0,83 ± 0,02	0,78-0,88	0,81 ± 0,02	0,78-0,85	0,49	0,487
		Relative	0,88 ± 0,03	0,82-0,94	0,84 ± 0,02	0,80-0,88	1,72	0,192
Beta2 (20-35 Hz)	N1	Absolute	0,75 ± 0,03	0,68-0,81	0,78 ± 0,02	0,74-0,82	0,71	0,400
		Relative	0,82 ± 0,04	0,74-0,90	0,86 ± 0,03	0,81-0,92	0,59	0,444
	N2	Absolute	0,77 ± 0,03	0,70-0,83	0,79 ± 0,02	0,74-0,83	0,22	0,638
		Relative	0,72 ± 0,04	0,64-0,80	0,78 ± 0,03	0,73-0,83	1,65	0,201
	N3	Absolute	0,75 ± 0,03	0,69-0,82	0,77 ± 0,02	0,73-0,81	0,17	0,680
		Relative	0,68 ± 0,04	0,60-0,76	0,74 ± 0,03	0,69-0,80	1,62	0,206
	REM	Absolute	0,88 ± 0,03	0,82-0,95	0,92 ± 0,02	0,88-0,96	1,05	0,307
		Relative	0,93 ± 0,04	0,85-1,01	0,96 ± 0,03	0,90-1,01	0,28	0,601
Gamma (35-60 Hz)	N1	Absolute	0,83 ± 0,03	0,77-0,89	0,88 ± 0,02	0,84-0,92	2,00	0,161
		Relative	0,92 ± 0,04	0,83-1,01	0,98 ± 0,03	0,92-1,04	1,01	0,316
	N2	Absolute	0,80 ± 0,03	0,74-0,86	0,88 ± 0,02	0,84-0,92	4,18	**0,044**[a]
		Relative	0,75 ± 0,04	0,67-0,84	0,87 ± 0,03	0,81-0,93	5,02	**0,027**[a]
	N3	Absolute	0,79 ± 0,03	0,73-0,85	0,87 ± 0,02	0,83-0,91	5,45	**0,022**[a]
		Relative	0,71 ± 0,04	0,62-0,80	0,86 ± 0,03	0,80-0,92	**7,68**	**0,006**[b]
	REM	Absolute	0,79 ± 0,03	0,74-0,85	0,85 ± 0,02	0,82-0,89	2,84	0,095
		Relative	0,84 ± 0,04	0,75-0,93	0,89 ± 0,03	0,83-0,95	0,83	0,365

[a]$p < 0.05$; [b]$p \leq 0.006$.

Table S4. – Mean left fronto-parietal (F3/P3) asymmetry of good sleepers and insomnia sufferers.

.

Frequency	Stage	Type	GS		INS		*F*	Sig.
			Mean ± SD	95% CI	Mean ± SD	95% CI		
Slow waves (0-1 Hz)	N1	Absolute	1,65 ± 0,31	1,03-2,26	1,92 ± 0,21	1,50-2,33	0,50	0,481
		Relative	1,27 ± 0,08	1,11-1,43	1,31 ± 0,05	1,21-1,42	0,20	0,657
	N2	Absolute	2,38 ± 0,31	1,76-2,99	1,67 ± 0,21	1,26-2,09	3,51	0,063
		Relative	1,20 ± 0,08	1,04-1,36	1,14 ± 0,05	1,03-1,24	0,46	0,498
	N3	Absolute	1,46 ± 0,31	0,85-2,08	1,24 ± 0,21	0,82-1,67	0,34	0,560
		Relative	0,80 ± 0,08	0,64-0,96	0,83 ± 0,06	0,72-0,94	0,11	0,738
	REM	Absolute	1,45 ± 0,31	0,83-2,06	1,60 ± 0,21	1,18-2,01	0,16	0,692
		Relative	1,17 ± 0,08	1,01-1,33	1,23 ± 0,05	1,13-1,34	0,42	0,519
Delta (1-4 Hz)	N1	Absolute	1,36 ± 0,13	0,92-1,80	1,63 ± 0,15	1,33-1,92	1,03	0,314
		Relative	1,11 ± 0,11	0,69-1,53	1,48 ± 0,14	1,20-1,77	2,18	0,144
	N2	Absolute	1,65 ± 0,13	1,21-2,09	1,83 ± 0,15	1,54-2,13	0,46	0,498
		Relative	1,11 ± 0,11	0,69-1,53	1,37 ± 0,14	1,09-1,65	1,05	0,309
	N3	Absolute	1,77 ± 0,13	1,33-2,21	1,95 ± 0,15	1,65-2,24	0,44	0,507
		Relative	1,24 ± 0,11	0,82-1,65	1,32 ± 0,14	1,03-1,60	0,10	0,750
	REM	Absolute	1,22 ± 0,13	0,96-1,48	1,81 ± 0,15	1,51-2,11	**8,17**	**0,005**[b]
		Relative	1,14 ± 0,11	0,72-1,56	1,31 ± 0,14	1,02-1,59	0,44	0,508
Theta (4-7 Hz)	N1	Absolute	1,01 ± 0,12	0,62-1,40	1,21 ± 0,10	0,95-1,47	0,76	0,387
		Relative	0,83 ± 0,38	0,08-1,59	1,43 ± 0,26	0,92-1,95	1,69	0,195
	N2	Absolute	1,01 ± 0,12	0,62-1,39	1,14 ± 0,10	0,87-1,40	0,30	0,584
		Relative	0,69 ± 0,38	-0,06-1,44	0,93 ± 0,26	0,43-1,44	0,29	0,594
	N3	Absolute	1,15 ± 0,12	0,76-1,54	1,31 ± 0,10	1,04-1,57	0,46	0,502
		Relative	0,83 ± 0,38	0,08-1,58	0,94 ± 0,26	0,42-1,46	0,06	0,807
	REM	Absolute	1,06 ± 0,12	0,82-1,30	1,42 ± 0,10	1,32-1,72	**7,95**	**0,006**[b]
		Relative	1,05 ± 0,38	0,30-1,80	1,20 ± 0,26	0,70-1,71	0,09	0,759
Alpha (7-11 Hz)	N1	Absolute	0,81 ± 0,16	0,49-1,14	0,79 ± 0,11	0,57-1,01	0,01	0,911
		Relative	0,67 ± 0,22	0,23-1,11	0,86 ± 0,15	0,56-1,16	0,51	0,475
	N2	Absolute	0,94 ± 0,16	0,61-1,27	0,99 ± 0,11	0,77-1,21	0,06	0,800
		Relative	0,64 ± 0,22	0,20-1,08	0,81 ± 0,15	0,52-1,11	0,43	0,516
	N3	Absolute	1,43 ± 0,16	1,10-1,76	1,52 ± 0,11	1,30-1,74	0,19	0,661
		Relative	1,03 ± 0,22	0,60-1,47	1,12 ± 0,15	0,82-1,42	0,10	0,757
	REM	Absolute	0,85 ± 0,16	0,52-1,18	0,92 ± 0,11	0,70-1,14	0,13	0,720

		Relative	0,78 ± 0,22	0,34-1,22	0,82 ± 0,15	0,52-1,11	0,02	0,896
Sigma (11-14 Hz)	N1	Absolute	0,89 ± 0,14	0,60-1,17	0,97 ± 0,10	0,78-1,16	0,21	0,645
		Relative	0,73 ± 0,21	0,32-1,15	0,99 ± 0,14	0,70-1,27	0,99	0,321
	N2	Absolute	1,12 ± 0,14	0,84-1,41	1,21 ± 0,10	1,02-1,40	0,26	0,609
		Relative	0,75 ± 0,21	0,34-1,17	1,01 ± 0,14	0,73-1,29	1,06	0,306
	N3	Absolute	1,38 ± 0,14	1,10-1,67	1,48 ± 0,10	1,29-1,67	0,34	0,562
		Relative	0,97 ± 0,21	0,56-1,39	1,15 ± 0,14	0,86-1,43	0,46	0,497
	REM	Absolute	0,76 ± 0,14	0,48-1,05	0,81 ± 0,10	0,62-1,00	0,09	0,766
		Relative	0,69 ± 0,21	0,27-1,10	0,71 ± 0,14	0,43-0,99	0,01	0,933
Beta1 (14-20 Hz)	N1	Absolute	0,96 ± 0,07	0,81-1,11	1,02 ± 0,05	0,92-1,12	0,53	0,470
		Relative	0,67 ± 0,12	0,43-0,91	0,99 ± 0,07	0,85-1,13	4.25	**0,040**[a]
	N2	Absolute	0,68 ± 0,07	0,54-0,83	0,73 ± 0,05	0,63-0,83	0,28	0,602
		Relative	0,46 ± 0,12	0,23-0,70	0,59 ± 0,07	0,43-0,74	0,72	0,396
	N3	Absolute	0,71 ± 0,07	0,56-0,86	0,75 ± 0,05	0,65-0,85	0,18	0,675
		Relative	0,52 ± 0,12	0,29-0,75	0,59 ± 0,07	0,43-0,75	0,21	0,644
	REM	Absolute	1,05 ± 0,07	0,90-1,20	1,01 ± 0,05	0,91-1,11	0,19	0,666
		Relative	0,94 ± 0,12	0,70-1,17	0,87 ± 0,07	0,71-1,03	0,23	0,634
Beta2 (20-35 Hz)	N1	Absolute	1,24 ± 0,10	1,04-1,45	1,31 ± 0,07	1,17-1,44	0,26	0,610
		Relative	1,04 ± 0,13	0,78-1,31	1,23 ± 0,09	1,05-1,41	1,36	0,246
	N2	Absolute	1,07 ± 0,10	0,87-1,28	1,06 ± 0,07	0,92-1,20	0,01	0,935
		Relative	0,74 ± 0,13	0,48-1,01	0,83 ± 0,09	0,65-1,00	0,26	0,614
	N3	Absolute	1,04 ± 0,10	0,83-1,24	1,01 ± 0,07	0,87-1,15	0,04	0,841
		Relative	0,77 ± 0,13	0,50-1,03	0,80 ± 0,09	0,62-0,98	0,04	0,844
	REM	Absolute	1,47 ± 0,10	1,27-1,68	1,53 ± 0,07	1,40-1,67	0,23	0,630
		Relative	1,28 ± 0,13	1,02-1,54	1,31 ± 0,09	1,13-1,49	0,04	0,850
Gamma (35-60 Hz)	N1	Absolute	1,21 ± 0,08	1,06-1,37	1,29 ± 0,05	1,18-1,39	0,62	0,432
		Relative	1,02 ± 0,10	0,82-1,21	1,17 ± 0,07	1,03-1,30	1,54	0,216
	N2	Absolute	1,13 ± 0,08	0,98-1,28	1,13 ± 0,05	1,03-1,23	0,00	1,000
		Relative	0,82 ± 0,10	0,62-1,01	0,88 ± 0,07	0,75-1,01	0,28	0,595
	N3	Absolute	1,12 ± 0,08	0,97-1,28	1,11 ± 0,05	1,01-1,22	0,02	0,902
		Relative	0,87 ± 0,10	0,68-1,07	0,89 ± 0,07	0,76-1,03	0,03	0,865
	REM	Absolute	1,17 ± 0,08	1,02-1,32	1,17 ± 0,05	1,07-1,28	0,00	0,961
		Relative	1,04 ± 0,10	0,84-1,23	1,01 ± 0,07	0,88-1,15	0,03	0,861

[a] $p < 0.05$; [b] $p \leq 0.006$.

Table S5. – Mean right fronto-parietal (F4/P4) asymmetry of good sleepers and insomnia sufferers.

Frequency	Stage	Type	GS		INS		*F*	Sig.
			Mean ± SD	95% CI	Mean ± SD	95% CI		
Slow waves (0-1 Hz)	N1	Absolute	2,03 ± 0,29	1,46-2,61	2,16 ± 0,20	1,77-2,56	0,14	0,711
		Relative	1,49 ± 0,07	1,34-1,63	1,48 ± 0,05	1,39-1,58	0,00	0,978
	N2	Absolute	2,13 ± 0,29	1,55-2,71	1,71 ± 0,20	1,32-2,10	1,42	0,236
		Relative	1,22 ± 0,07	1,08-1,36	1,18 ± 0,05	1,08-1,27	0,24	0,626
	N3	Absolute	1,48 ± 0,29	0,91-2,06	1,23 ± 0,20	0,83-1,62	0,52	0,470
		Relative	0,85 ± 0,07	0,71-0,99	0,86 ± 0,05	0,76-0,96	0,01	0,905
	REM	Absolute	2,04 ± 0,29	1,47-2,62	2,24 ± 0,20	1,85-2,63	0,32	0,575
		Relative	1,42 ± 0,07	1,27-1,56	1,50 ± 0,05	1,40-1,59	0,84	0,362
Delta (1-4 Hz)	N1	Absolute	1,31 ± 0,07	1,19-1,44	1,41 ± 0,04	1,32-1,50	1,47	0,227
		Relative	1,04 ± 0,05	0,95-1,14	1,16 ± 0,03	1,10-1,23	4,21	**0,041**[a]
	N2	Absolute	1,61 ± 0,07	1,49-1,74	1,56 ± 0,04	1,47-1,65	0,48	0,489
		Relative	1,11 ± 0,05	1,02-1,21	1,21 ± 0,03	1,15-1,27	2,82	0,094
	N3	Absolute	1,73 ± 0,07	1,60-1,86	1,60 ± 0,04	1,52-1,69	2,45	0,120
		Relative	1,18 ± 0,05	1,09-1,28	1,20 ± 0,03	1,14-1,27	0,08	0,774
	REM	Absolute	1,41 ± 0,07	1,28-1,54	1,51 ± 0,04	1,43-1,60	1,80	0,181
		Relative	1,05 ± 0,05	0,96-1,15	1,05 ± 0,03	0,98-1,11	0,01	0,932
Theta (4-7 Hz)	N1	Absolute	0,98 ± 0,04	0,90-1,06	0,99 ± 0,03	0,94-1,05	0,09	0,764
		Relative	0,79 ± 0,06	0,68-0,90	0,83 ± 0,04	0,75-0,91	0,33	0,567
	N2	Absolute	1,00 ± 0,04	0,92-1,08	0,97 ± 0,03	0,92-1,03	0,35	0,557
		Relative	0,70 ± 0,06	0,59-0,81	0,79 ± 0,04	0,72-0,87	1,87	0,172
	N3	Absolute	1,12 ± 0,04	1,04-1,21	1,09 ± 0,03	1,03-1,14	0,63	0,431
		Relative	0,79 ± 0,06	0,68-0,91	0,84 ± 0,04	0,76-0,92	0,47	0,494
	REM	Absolute	1,16 ± 0,04	1,08-1,24	1,15 ± 0,03	1,10-1,21	0,00	0,953
		Relative	0,89 ± 0,06	0,78-1,01	0,85 ± 0,04	0,77-0,92	0,45	0,505
Alpha (7-11 Hz)	N1	Absolute	0,79 ± 0,05	0,69-0,89	0,70 ± 0,03	0,63-0,76	2,44	0,121
		Relative	0,63 ± 0,06	0,52-0,75	0,58 ± 0,04	0,50-0,66	0,56	0,453
	N2	Absolute	0,94 ± 0,05	0,84-1,04	0,87 ± 0,03	0,81-0,94	1,13	0,290
		Relative	0,66 ± 0,06	0,54-0,77	0,72 ± 0,04	0,64-0,80	0,89	0,347
	N3	Absolute	1,33 ± 0,05	1,23-1,43	1,25 ± 0,03	1,18-1,31	1,79	0,184
		Relative	0,94 ± 0,06	0,83-1,05	0,96 ± 0,04	0,88-1,04	0,12	0,729
	REM	Absolute	0,80 ± 0,05	0,70-0,90	0,75 ± 0,03	0,69-0,82	0,68	0,412
		Relative	0,62 ± 0,06	0,51-0,73	0,54 ± 0,04	0,47-0,62	1,17	0,281

Sigma (11-14 Hz)	N1	Absolute	0,85 ± 0,07	0,72-0,98	0,85 ± 0,05	0,76-0,94	0,00	0,958
		Relative	0,68 ± 0,06	0,56-0,80	0,73 ± 0,04	0,65-0,81	0,41	0,525
	N2	Absolute	1,13 ± 0,07	1,00-1,26	1,07 ± 0,04	0,98-1,16	0,47	0,496
		Relative	0,79 ± 0,06	0,67-0,91	0,86 ± 0,04	0,78-0,93	0,80	0,371
	N3	Absolute	1,32 ± 0,07	1,19-1,45	1,32 ± 0,05	1,23-1,41	0,00	0,966
		Relative	0,91 ± 0,06	0,80-1,03	1,02 ± 0,04	0,94-1,10	2,23	0,138
	REM	Absolute	0,72 ± 0,07	0,59-0,86	0,71 ± 0,04	0,62-0,80	0,02	0,889
		Relative	0,55 ± 0,06	0,44-0,67	0,52 ± 0,04	0,44-0,60	0,24	0,628
Beta1 (14-20 Hz)	N1	Absolute	0,92 ± 0,04	0,85-0,99	0,91 ± 0,02	0,87-0,96	0,02	0,889
		Relative	0,74 ± 0,05	0,64-0,85	0,78 ± 0,04	0,71-0,85	0,29	0,594
	N2	Absolute	0,72 ± 0,04	0,64-0,79	0,69 ± 0,02	0,64-0,74	0,38	0,539
		Relative	0,50 ± 0,05	0,39-0,60	0,58 ± 0,04	0,51-0,65	1,68	0,196
	N3	Absolute	0,74 ± 0,04	0,67-0,81	0,73 ± 0,02	0,68-0,78	0,04	0,851
		Relative	0,52 ± 0,05	0,42-0,63	0,57 ± 0,04	0,50-0,64	0,62	0,431
	REM	Absolute	0,99 ± 0,04	0,92-1,06	0,91 ± 0,02	0,87-0,96	3,26	0,074
		Relative	0,76 ± 0,05	0,66-0,86	0,67 ± 0,04	0,60-0,74	1,91	0,169
Beta2 (20-35 Hz)	N1	Absolute	1,14 ± 0,10	0,95-1,33	1,23 ± 0,07	1,10-1,36	0,54	0,463
		Relative	0,94 ± 0,09	0,76-1,12	1,04 ± 0,06	0,92-1,16	0,90	0,345
	N2	Absolute	1,00 ± 0,10	0,81-1,19	0,99 ± 0,07	0,86-1,12	0,01	0,938
		Relative	0,70 ± 0,09	0,52-0,88	0,82 ± 0,06	0,70-0,94	1,24	0,267
	N3	Absolute	0,96 ± 0,10	0,77-1,16	0,94 ± 0,07	0,81-1,07	0,03	0,864
		Relative	0,69 ± 0,09	0,51-0,86	0,74 ± 0,06	0,61-0,86	0,22	0,643
	REM	Absolute	1,43 ± 0,10	1,24-1,62	1,47 ± 0,07	1,34-1,60	0,14	0,707
		Relative	1,08 ± 0,09	0,90-1,25	1,08 ± 0,06	0,96-1,20	0,00	0,991
Gamma (35-60 Hz)	N1	Absolute	1,06 ± 0,06	0,94-1,17	1,15 ± 0,04	1,07-1,23	1,90	0,172
		Relative	0,86 ± 0,07	0,72-1,00	0,98 ± 0,05	0,88-1,07	1,86	0,174
	N2	Absolute	1,02 ± 0,06	0,91-1,13	1,04 ± 0,04	0,97-1,12	0,12	0,725
		Relative	0,72 ± 0,07	0,58-0,86	0,87 ± 0,05	0,77-0,96	2,96	0,087
	N3	Absolute	1,01 ± 0,06	0,90-1,13	0,98 ± 0,04	0,90-1,05	0,28	0,595
		Relative	0,72 ± 0,07	0,58-0,87	0,76 ± 0,05	0,66-0,85	0,14	0,710
	REM	Absolute	1,09 ± 0,06	0,97-1,20	1,09 ± 0,04	1,02-1,17	0,00	0,944
		Relative	0,84 ± 0,07	0,70-0,98	0,82 ± 0,05	0,72-0,91	0,09	0,763

[a]$p < 0.05$; [b]$p \leq 0.006$

Table S6. – Mean mid fronto-parietal (Fz/Pz) asymmetry of good sleepers and insomnia sufferers.

Frequency	Stage	Type	GS		INS		*F*	Sig.
			Mean ± SD	95% CI	Mean ± SD	95% CI		
Slow waves (0-1 Hz)	N1	Absolute	1,33 ± 0,12	1,10-1,56	1,46 ± 0,08	1,30-1,62	0,85	0,358
		Relative	1,18 ± 0,05	1,09-1,28	1,20 ± 0,03	1,14-1,27	0,11	0,742
	N2	Absolute	1,42 ± 0,12	1,19-1,65	1,34 ± 0,08	1,18-1,49	0,37	0,542
		Relative	1,08 ± 0,05	0,99-1,18	1,07 ± 0,03	1,00-1,13	0,10	0,755
	N3	Absolute	1,03 ± 0,12	0,80-1,26	0,98 ± 0,08	0,82-1,14	0,10	0,754
		Relative	0,78 ± 0,05	0,69-0,87	0,79 ± 0,03	0,72-0,85	0,03	0,870
	REM	Absolute	1,43 ± 0,12	1,20-1,66	1,52 ± 0,08	1,36-1,67	0,34	0,560
		Relative	1,18 ± 0,05	1,09-1,27	1,23 ± 0,03	1,17-1,29	0,73	0,395
Delta (1-4 Hz)	N1	Absolute	1,29 ± 0,07	1,16-1,42	1,42 ± 0,04	1,33-1,50	2,68	0,104
		Relative	1,15 ± 0,03	1,09-1,21	1,27 ± 0,02	1,22-1,31	**9,64**	**0,002**[b]
	N2	Absolute	1,50 ± 0,07	1,37-1,63	1,51 ± 0,04	1,42-1,60	0,01	0,903
		Relative	1,17 ± 0,03	1,11-1,23	1,22 ± 0,02	1,18-1,26	1,51	0,221
	N3	Absolute	1,57 ± 0,07	1,45-1,70	1,52 ± 0,04	1,43-1,61	0,51	0,478
		Relative	1,23 ± 0,03	1,17-1,29	1,23 ± 0,02	1,18-1,27	0,01	0,923
	REM	Absolute	1,24 ± 0,07	1,11-1,37	1,32 ± 0,04	1,23-1,40	1,01	0,316
		Relative	1,04 ± 0,03	0,98-1,11	1,10 ± 0,02	1,06-1,14	1,89	0,171
Theta (4-7 Hz)	N1	Absolute	0,96 ± 0,05	0,87-1,05	0,98 ± 0,03	0,92-1,05	0,18	0,674
		Relative	0,86 ± 0,04	0,79-0,93	0,90 ± 0,02	0,85-0,95	0,64	0,425
	N2	Absolute	0,95 ± 0,05	0,86-1,04	0,93 ± 0,03	0,87-0,99	0,13	0,722
		Relative	0,75 ± 0,04	0,67-0,82	0,76 ± 0,02	0,72-0,81	0,21	0,644
	N3	Absolute	1,07 ± 0,05	0,98-1,16	1,04 ± 0,03	0,98-1,10	0,22	0,637
		Relative	0,85 ± 0,04	0,78-0,92	0,87 ± 0,02	0,82-0,92	0,20	0,657
	REM	Absolute	1,16 ± 0,05	1,07-1,25	1,15 ± 0,03	1,09-1,21	0,06	0,800
		Relative	1,00 ± 0,04	0,93-1,07	0,96 ± 0,02	0,92-1,01	0,76	0,383
Alpha (7-11 Hz)	N1	Absolute	0,76 ± 0,06	0,64-0,88	0,69 ± 0,04	0,61-0,77	1,02	0,314
		Relative	0,68 ± 0,05	0,59-0,78	0,62 ± 0,03	0,56-0,69	1,00	0,319
	N2	Absolute	0,87 ± 0,06	0,76-0,99	0,85 ± 0,04	0,77-0,93	0,13	0,718
		Relative	0,69 ± 0,05	0,59-0,78	0,70 ± 0,03	0,64-0,76	0,04	0,848
	N3	Absolute	1,28 ± 0,06	1,17-1,40	1,24 ± 0,04	1,16-1,32	0,34	0,564
		Relative	1,03 ± 0,05	0,93-1,13	1,05 ± 0,03	0,98-1,11	0,08	0,779
	REM	Absolute	0,81 ± 0,06	0,69-0,93	0,78 ± 0,04	0,70-0,86	0,11	0,738
		Relative	0,68 ± 0,05	0,59-0,78	0,65 ± 0,03	0,59-0,72	0,24	0,622

Sigma (11-14 Hz)	N1	Absolute	0,85 ± 0,07	0,71-0,99	0,87 ± 0,05	0,78-0,97	0,07	0,791
		Relative	0,76 ± 0,05	0,66-0,87	0,80 ± 0,04	0,73-0,87	0,39	0,533
	N2	Absolute	0,94 ± 0,07	0,80-1,08	0,91 ± 0,05	0,81-1,00	0,11	0,738
		Relative	0,74 ± 0,05	0,64-0,84	0,74 ± 0,04	0,67-0,81	0,00	0,975
	N3	Absolute	1,14 ± 0,07	1,00-1,28	1,13 ± 0,05	1,03-1,22	0,03	0,854
		Relative	0,88 ± 0,05	0,78-0,99	0,94 ± 0,04	0,86-1,01	0,71	0,400
	REM	Absolute	0,73 ± 0,07	0,59-0,87	0,74 ± 0,05	0,64-0,83	0,00	0,949
		Relative	0,62 ± 0,05	0,52-0,73	0,62 ± 0,04	0,55-0,69	0,01	0,943
Beta1 (14-20 Hz)	N1	Absolute	0,89 ± 0,04	0,81-0,97	0,90 ± 0,03	0,84-0,95	0,04	0,838
		Relative	0,80 ± 0,03	0,74-0,87	0,83 ± 0,02	0,78-0,87	0,41	0,523
	N2	Absolute	0,58 ± 0,04	0,50-0,66	0,58 ± 0,03	0,52-0,63	0,01	0,935
		Relative	0,45 ± 0,03	0,39-0,52	0,47 ± 0,02	0,43-0,52	0,30	0,585
	N3	Absolute	0,59 ± 0,04	0,51-0,67	0,60 ± 0,03	0,55-0,66	0,03	0,866
		Relative	0,48 ± 0,03	0,41-0,54	0,51 ± 0,02	0,46-0,55	0,66	0,417
	REM	Absolute	1,00 ± 0,04	0,91-1,08	0,93 ± 0,03	0,88-0,99	1,68	0,198
		Relative	0,84 ± 0,03	0,78-0,91	0,79 ± 0,02	0,74-0,83	2,05	0,154
Beta2 (20-35 Hz)	N1	Absolute	1,10 ± 0,09	0,92-1,28	1,18 ± 0,06	1,06-1,30	0,57	0,451
		Relative	1,00 ± 0,08	0,85-1,16	1,09 ± 0,05	0,99-1,20	0,91	0,342
	N2	Absolute	0,93 ± 0,09	0,76-1,11	0,95 ± 0,06	0,83-1,07	0,01	0,919
		Relative	0,74 ± 0,08	0,59-0,90	0,78 ± 0,05	0,68-0,89	0,19	0,662
	N3	Absolute	0,91 ± 0,09	0,73-1,09	0,90 ± 0,06	0,78-1,02	0,01	0,931
		Relative	0,74 ± 0,08	0,58-0,89	0,76 ± 0,05	0,65-0,86	0,05	0,818
	REM	Absolute	1,32 ± 0,09	1,14-1,49	1,40 ± 0,06	1,28-1,52	0,58	0,449
		Relative	1,10 ± 0,08	0,95-1,26	1,19 ± 0,05	1,09-1,29	0,81	0,371
Gamma (35-60 Hz)	N1	Absolute	1,00 ± 0,07	0,87-1,13	1,13 ± 0,04	1,04-1,21	2,59	0,111
		Relative	0,92 ± 0,10	0,72-1,11	1,07 ± 0,07	0,93-1,20	1,55	0,217
	N2	Absolute	0,95 ± 0,07	0,82-1,08	1,05 ± 0,04	0,96-1,13	1,40	0,241
		Relative	0,76 ± 0,10	0,57-0,96	0,93 ± 0,07	0,80-1,06	1,94	0,168
	N3	Absolute	0,94 ± 0,07	0,81-1,07	0,95 ± 0,04	0,87-1,04	0,04	0,841
		Relative	0,76 ± 0,10	0,57-0,96	0,86 ± 0,07	0,73-1,00	0,71	0,402
	REM	Absolute	0,99 ± 0,07	0,86-1,12	1,07 ± 0,04	0,98-1,16	1,04	0,310
		Relative	0,85 ± 0,10	0,66-1,05	0,95 ± 0,07	0,82-1,08	0,71	0,401

[a]$p < 0.05$; [b]$p \leq 0.006$.

Table S7. – Mean left fronto-occipital (F3/O1) asymmetry of good sleepers and insomnia sufferers.

Frequency	Stage	Type	GS		INS		*F*	Sig.
			Mean ± SD	95% CI	Mean ± SD	95% CI		
Slow waves (0-1 Hz)	N1	Absolute	1,70 ± 0,27	1,17-2,23	1,86 ± 0,18	1,50-2,22	0,23	0,633
		Relative	1,22 ± 0,07	1,08-1,36	1,28 ± 0,05	1,19-1,38	0,58	0,447
	N2	Absolute	3,09 ± 0,27	2,56-3,62	2,19 ± 0,18	1,83-2,55	**7,85**	**0,006[b]**
		Relative	1,28 ± 0,07	1,14-1,42	1,21 ± 0,05	1,12-1,31	0,52	0,471
	N3	Absolute	1,90 ± 0,27	1,37-2,43	1,56 ± 0,18	1,20-1,93	1,11	0,293
		Relative	0,75 ± 0,07	0,61-0,89	0,79 ± 0,05	0,69-0,89	0,19	0,662
	REM	Absolute	1,60 ± 0,27	1,07-2,13	1,67 ± 0,18	1,31-2,03	0,05	0,832
		Relative	1,13 ± 0,07	0,99-1,27	1,24 ± 0,05	1,14-1,33	1,77	0,186
Delta (1-4 Hz)	N1	Absolute	1,59 ± 0,14	1,31-1,88	1,72 ± 0,10	1,53-1,92	0,56	0,458
		Relative	1,20 ± 0,04	1,13-1,28	1,29 ± 0,03	1,24-1,34	4,01	**0,047[a]**
	N2	Absolute	2,43 ± 0,14	2,15-2,72	2,24 ± 0,10	2,05-2,43	1,29	0,259
		Relative	1,19 ± 0,04	1,12-1,27	1,28 ± 0,03	1,23-1,33	3,87	0,051
	N3	Absolute	2,91 ± 0,14	2,62-3,19	2,63 ± 0,10	2,44-2,83	2,45	0,121
		Relative	1,37 ± 0,04	1,30-1,45	1,35 ± 0,03	1,29-1,40	0,32	0,572
	REM	Absolute	1,55 ± 0,14	1,26-1,83	1,48 ± 0,10	1,29-1,67	0,16	0,694
		Relative	1,12 ± 0,04	1,04-1,19	1,15 ± 0,03	1,10-1,20	0,42	0,518
Theta (4-7 Hz)	N1	Absolute	1,01 ± 0,09	0,84-1,19	0,97 ± 0,06	0,85-1,09	0,17	0,681
		Relative	0,77 ± 0,04	0,69-0,85	0,75 ± 0,03	0,70-0,81	0,15	0,702
	N2	Absolute	0,98 ± 0,09	0,81-1,16	0,88 ± 0,06	0,76-1,00	0,92	0,341
		Relative	0,48 ± 0,04	0,41-0,56	0,52 ± 0,03	0,46-0,57	0,49	0,485
	N3	Absolute	1,22 ± 0,09	1,05-1,39	1,09 ± 0,06	0,98-1,21	1,41	0,239
		Relative	0,59 ± 0,04	0,52-0,67	0,58 ± 0,03	0,53-0,63	0,08	0,779
	REM	Absolute	1,44 ± 0,09	1,26-1,61	1,17 ± 0,06	1,05-1,29	6,36	**0,014[a]**
		Relative	1,03 ± 0,04	0,96-1,11	0,93 ± 0,03	0,88-0,98	4,91	**0,028[a]**
Alpha (7-11 Hz)	N1	Absolute	0,85 ± 0,10	0,66-1,04	0,70 ± 0,07	0,57-0,83	1,66	0,201
		Relative	0,65 ± 0,05	0,55-0,74	0,56 ± 0,03	0,49-0,62	2,53	0,114
	N2	Absolute	1,13 ± 0,10	0,94-1,33	0,95 ± 0,07	0,82-1,08	2,45	0,120
		Relative	0,58 ± 0,05	0,48-0,67	0,57 ± 0,03	0,50-0,63	0,04	0,840
	N3	Absolute	1,82 ± 0,10	1,62-2,01	1,52 ± 0,07	1,39-1,65	6,32	**0,013[a]**
		Relative	0,92 ± 0,05	0,82-1,01	0,80 ± 0,03	0,73-0,86	3,96	**0,048[a]**
	REM	Absolute	0,90 ± 0,10	0,71-1,09	0,74 ± 0,07	0,61-0,87	1,79	0,183
		Relative	0,65 ± 0,05	0,55-0,74	0,59 ± 0,03	0,53-0,66	0,79	0,376

Sigma (11-14 Hz)	N1	Absolute	1,00 ± 0,15	0,71-1,29	0,98 ± 0,10	0,78-1,17	0,02	0,895
		Relative	0,77 ± 0,07	0,63-0,92	0,79 ± 0,05	0,70-0,89	0,05	0,831
	N2	Absolute	1,89 ± 0,15	1,59-2,18	1,59 ± 0,10	1,39-1,78	2,84	0,095
		Relative	0,92 ± 0,07	0,78-1,07	0,95 ± 0,05	0,85-1,05	0,09	0,769
	N3	Absolute	2,25 ± 0,15	1,96-2,55	1,95 ± 0,10	1,75-2,15	2,85	0,094
		Relative	1,03 ± 0,07	0,88-1,17	1,08 ± 0,05	0,99-1,18	0,45	0,502
	REM	Absolute	0,80 ± 0,15	0,51-1,10	0,69 ± 0,10	0,50-0,89	0,36	0,550
		Relative	0,58 ± 0,07	0,44-0,72	0,57 ± 0,05	0,47-0,66	0,02	0,893
Beta1 (14-20 Hz)	N1	Absolute	1,21 ± 0,08	1,05-1,37	1,14 ± 0,05	1,03-1,24	0,53	0,468
		Relative	0,94 ± 0,05	0,84-1,03	0,93 ± 0,03	0,86-0,99	0,02	0,880
	N2	Absolute	1,10 ± 0,08	0,94-1,25	0,95 ± 0,05	0,84-1,05	2,38	0,127
		Relative	0,55 ± 0,05	0,45-0,65	0,58 ± 0,03	0,52-0,65	0,35	0,554
	N3	Absolute	1,04 ± 0,08	0,88-1,19	0,96 ± 0,05	0,85-1,07	0,64	0,425
		Relative	0,52 ± 0,05	0,42-0,61	0,55 ± 0,03	0,48-0,61	0,27	0,605
	REM	Absolute	1,25 ± 0,08	1,09-1,41	1,02 ± 0,05	0,91-1,12	6,02	**0,016**[a]
		Relative	0,88 ± 0,05	0,78-0,97	0,86 ± 0,03	0,79-0,92	0,09	0,763
Beta2 (20-35 Hz)	N1	Absolute	1,50 ± 0,18	1,14-1,85	1,41 ± 0,12	1,18-1,65	0,15	0,701
		Relative	1,13 ± 0,11	0,92-1,35	1,18 ± 0,07	1,04-1,33	0,13	0,717
	N2	Absolute	1,18 ± 0,18	0,83-1,53	1,05 ± 0,12	0,81-1,28	0,38	0,539
		Relative	0,61 ± 0,11	0,40-0,83	0,65 ± 0,07	0,50-0,79	0,07	0,791
	N3	Absolute	1,09 ± 0,18	0,74-1,44	1,01 ± 0,12	0,77-1,25	0,16	0,694
		Relative	0,56 ± 0,11	0,34-0,77	0,57 ± 0,07	0,42-0,71	0,01	0,941
	REM	Absolute	2,04 ± 0,18	1,69-2,39	1,71 ± 0,12	1,48-1,95	2,34	0,129
		Relative	1,33 ± 0,11	1,11-1,54	1,47 ± 0,07	1,32-1,61	1,14	0,288
Gamma (35-60 Hz)	N1	Absolute	1,19 ± 0,09	1,02-1,36	1,24 ± 0,06	1,13-1,36	0,25	0,616
		Relative	0,94 ± 0,08	0,79-1,10	1,04 ± 0,05	0,93-1,14	0,93	0,336
	N2	Absolute	1,12 ± 0,09	0,95-1,29	1,06 ± 0,06	0,95-1,18	0,27	0,608
		Relative	0,61 ± 0,08	0,45-0,76	0,67 ± 0,05	0,57-0,78	0,44	0,511
	N3	Absolute	1,10 ± 0,09	0,93-1,27	1,02 ± 0,06	0,91-1,14	0,58	0,449
		Relative	0,59 ± 0,08	0,44-0,75	0,60 ± 0,05	0,49-0,71	0,01	0,930
	REM	Absolute	1,23 ± 0,09	1,06-1,40	1,13 ± 0,06	1,01-1,24	0,96	0,329
		Relative	0,90 ± 0,08	0,75-1,06	0,97 ± 0,05	0,87-1,08	0,56	0,455

[a] $p < 0.05$; [b] $p \leq 0.006$.

Table S8. – Mean right fronto-occipital (F4/O2) asymmetry of good sleepers and insomnia sufferers.

Frequency	Stage	Type	GS		INS		*F*	Sig.
			Mean ± SD	95% CI	Mean ± SD	95% CI		
Slow waves (0-1 Hz)	N1	Absolute	2,23 ± 0,24	1,75-2,71	1,96 ± 0,17	1,63-2,28	0,87	0,351
		Relative	1,39 ± 0,07	1,25-1,53	1,38 ± 0,05	1,29-1,48	0,01	0,912
	N2	Absolute	2,78 ± 0,24	2,30-3,26	2,26 ± 0,16	1,93-2,58	3,24	0,074
		Relative	1,24 ± 0,07	1,10-1,38	1,21 ± 0,05	1,11-1,30	0,15	0,702
	N3	Absolute	1,89 ± 0,24	1,41-2,38	1,73 ± 0,17	1,40-2,06	0,33	0,566
		Relative	0,79 ± 0,07	0,65-0,93	0,82 ± 0,05	0,73-0,92	0,16	0,689
	REM	Absolute	2,51 ± 0,24	2,03-2,99	2,37 ± 0,16	2,05-2,70	0,23	0,632
		Relative	1,39 ± 0,07	1,25-1,53	1,48 ± 0,05	1,38-1,57	1,08	0,301
Delta (1-4 Hz)	N1	Absolute	1,58 ± 0,14	1,31-1,85	1,70 ± 0,09	1,52-1,89	0,56	0,455
		Relative	1,19 ± 0,04	1,11-1,28	1,25 ± 0,03	1,19-1,31	1,08	0,299
	N2	Absolute	2,47 ± 0,14	2,20-2,74	2,26 ± 0,09	2,08-2,45	1,57	0,212
		Relative	1,20 ± 0,04	1,11-1,28	1,28 ± 0,03	1,22-1,34	2,27	0,134
	N3	Absolute	3,03 ± 0,14	2,75-3,30	2,70 ± 0,09	2,52-2,89	3,79	0,054
		Relative	1,34 ± 0,04	1,25-1,43	1,33 ± 0,03	1,27-1,39	0,02	0,876
	REM	Absolute	1,80 ± 0,14	1,53-2,07	1,71 ± 0,09	1,52-1,89	0,31	0,577
		Relative	1,05 ± 0,04	0,96-1,13	1,11 ± 0,03	1,05-1,17	1,39	0,240
Theta (4-7 Hz)	N1	Absolute	1,00 ± 0,09	0,83-1,17	1,01 ± 0,06	0,89-1,12	0,00	0,989
		Relative	1,16 ± 0,14	0,89-1,44	0,74 ± 0,10	0,56-0,93	6,09	**0,014**[a]
	N2	Absolute	1,05 ± 0,09	0,88-1,22	0,95 ± 0,06	0,84-1,06	0,96	0,331
		Relative	0,50 ± 0,14	0,23-0,78	0,55 ± 0,09	0,36-0,74	0,08	0,774
	N3	Absolute	1,30 ± 0,09	1,13-1,47	1,17 ± 0,06	1,06-1,29	1,49	0,226
		Relative	0,58 ± 0,14	0,31-0,85	0,59 ± 0,10	0,40-0,78	0,01	0,938
	REM	Absolute	1,45 ± 0,09	1,28-1,62	1,21 ± 0,06	1,09-1,32	5,60	**0,020**[a]
		Relative	0,85 ± 0,14	0,57-1,12	0,84 ± 0,09	0,65-1,02	0,00	0,964
Alpha (7-11 Hz)	N1	Absolute	0,89 ± 0,09	0,70-1,07	0,77 ± 0,06	0,64-0,90	1,04	0,310
		Relative	1,18 ± 0,16	0,86-1,50	0,59 ± 0,11	0,37-0,81	**9,04**	**0,003**[b]
	N2	Absolute	1,24 ± 0,09	1,06-1,43	1,04 ± 0,06	0,92-1,17	3,10	0,081
		Relative	0,61 ± 0,16	0,29-0,93	0,61 ± 0,11	0,40-0,83	0,00	0,984
	N3	Absolute	1,84 ± 0,09	1,65-2,03	1,51 ± 0,06	1,39-1,64	**8,34**	**0,005**[b]
		Relative	0,84 ± 0,16	0,52-1,16	0,77 ± 0,11	0,55-0,99	0,11	0,737
	REM	Absolute	0,91 ± 0,09	0,72-1,09	0,77 ± 0,06	0,65-0,90	1,44	0,232
		Relative	0,53 ± 0,16	0,21-0,85	0,52 ± 0,11	0,31-0,74	0,00	0,952

Sigma (11-14 Hz)	N1	Absolute	1,05 ± 0,13	0,80-1,30	1,01 ± 0,08	0,84-1,18	0,05	0,819
		Relative	1,32 ± 0,18	0,98-1,67	0,77 ± 0,12	0,54-1,01	6,63	**0,010**[a]
	N2	Absolute	1,96 ± 0,13	1,71-2,21	1,68 ± 0,08	1,51-1,85	3,32	0,071
		Relative	0,96 ± 0,18	0,61-1,30	0,99 ± 0,12	0,76-1,23	0,03	0,873
	N3	Absolute	2,16 ± 0,13	1,90-2,41	1,95 ± 0,09	1,78-2,12	1,86	0,176
		Relative	0,93 ± 0,18	0,58-1,28	1,01 ± 0,12	0,76-1,25	0,13	0,722
	REM	Absolute	0,83 ± 0,13	0,58-1,08	0,75 ± 0,08	0,59-0,92	0,24	0,625
		Relative	0,49 ± 0,18	0,14-0,84	0,51 ± 0,12	0,28-0,75	0,01	0,918
Beta1 (14-20 Hz)	N1	Absolute	1,23 ± 0,08	1,08-1,38	1,16 ± 0,05	1,05-1,26	0,65	0,423
		Relative	1,54 ± 0,19	1,16-1,91	0,90 ± 0,13	0,64-1,16	**7,50**	**0,006**[b]
	N2	Absolute	1,16 ± 0,08	1,01-1,32	1,03 ± 0,05	0,93-1,13	2,13	0,148
		Relative	0,57 ± 0,19	0,19-0,95	0,62 ± 0,13	0,36-0,87	0,04	0,845
	N3	Absolute	1,09 ± 0,08	0,94-1,24	1,03 ± 0,05	0,92-1,13	0,47	0,493
		Relative	0,50 ± 0,19	0,12-0,88	0,55 ± 0,13	0,29-0,81	0,05	0,824
	REM	Absolute	1,27 ± 0,08	1,11-1,42	1,07 ± 0,05	0,97-1,17	4,53	**0,036**[a]
		Relative	0,74 ± 0,19	0,36-1,11	0,76 ± 0,13	0,51-1,02	0,01	0,913
Beta2 (20-35 Hz)	N1	Absolute	1,47 ± 0,20	1,07-1,87	1,51 ± 0,14	1,24-1,78	0,03	0,873
		Relative	1,58 ± 0,18	1,22-1,94	1,20 ± 0,13	0,96-1,45	2,91	0,089
	N2	Absolute	1,21 ± 0,20	0,81-1,61	1,11 ± 0,14	0,85-1,38	0,14	0,704
		Relative	0,61 ± 0,18	0,25-0,97	0,67 ± 0,12	0,43-0,91	0,09	0,769
	N3	Absolute	1,11 ± 0,20	0,71-1,51	1,04 ± 0,14	0,77-1,31	0,09	0,762
		Relative	0,52 ± 0,18	0,16-0,88	0,55 ± 0,13	0,30-0,80	0,03	0,868
	REM	Absolute	2,15 ± 0,20	1,75-2,55	1,90 ± 0,14	1,63-2,16	1,09	0,298
		Relative	1,18 ± 0,18	0,82-1,54	1,31 ± 0,12	1,07-1,56	0,37	0,542
Gamma (35-60 Hz)	N1	Absolute	1,18 ± 0,11	0,96-1,39	1,31 ± 0,07	1,17-1,45	1,06	0,306
		Relative	1,34 ± 0,15	1,04-1,63	1,04 ± 0,10	0,84-1,24	2,70	0,101
	N2	Absolute	1,15 ± 0,11	0,94-1,36	1,16 ± 0,07	1,01-1,30	0,00	0,951
		Relative	0,59 ± 0,15	0,30-0,89	0,71 ± 0,10	0,51-0,91	0,42	0,516
	N3	Absolute	1,13 ± 0,11	0,92-1,34	1,07 ± 0,07	0,93-1,22	0,17	0,681
		Relative	0,54 ± 0,15	0,25-0,83	0,58 ± 0,10	0,37-0,78	0,04	0,836
	REM	Absolute	1,31 ± 0,11	1,10-1,52	1,30 ± 0,07	1,16-1,44	0,00	0,945
		Relative	0,80 ± 0,15	0,51-1,09	0,93 ± 0,10	0,74-1,13	0,56	0,455

[a]$p < 0.05$; [b]$p \leq 0.006$.

Table S9. – Mean left centro-parietal (C3/P3) asymmetry of good sleepers and insomnia sufferers.

Frequency	Stage	Type	GS		INS		*F*	Sig.
			Mean ± SD	95% CI	Mean ± SD	95% CI		
Slow waves (0-1 Hz)	N1	Absolute	0,97 ± 0,05	0,88-1,07	0,90 ± 0,03	0,83-0,97	1,53	0,217
		Relative	0,94 ± 0,04	0,87-1,01	0,91 ± 0,02	0,86-0,95	0,70	0,403
	N2	Absolute	1,10 ± 0,05	1,00-1,20	1,01 ± 0,03	0,94-1,08	2,17	0,141
		Relative	0,99 ± 0,04	0,92-1,06	0,95 ± 0,02	0,90-0,99	1,18	0,278
	N3	Absolute	0,90 ± 0,05	0,81-1,00	0,97 ± 0,04	0,90-1,04	1,06	0,305
		Relative	0,87 ± 0,04	0,80-0,94	0,88 ± 0,02	0,83-0,93	0,04	0,833
	REM	Absolute	0,86 ± 0,05	0,76-0,96	0,87 ± 0,03	0,81-0,94	0,06	0,803
		Relative	0,88 ± 0,04	0,81-0,95	0,91 ± 0,02	0,87-0,96	0,75	0,389
Delta (1-4 Hz)	N1	Absolute	1,13 ± 0,14	0,85-1,40	1,27 ± 0,09	1,08-1,46	0,72	0,398
		Relative	1,10 ± 0,20	0,70-1,51	1,45 ± 0,14	1,18-1,72	2,02	0,159
	N2	Absolute	1,20 ± 0,14	0,92-1,47	1,33 ± 0,09	1,15-1,52	0,69	0,409
		Relative	1,09 ± 0,20	0,69-1,50	1,32 ± 0,14	1,05-1,60	0,89	0,349
	N3	Absolute	1,16 ± 0,14	0,88-1,43	1,36 ± 0,09	1,18-1,55	1,52	0,222
		Relative	1,13 ± 0,20	0,73-1,53	1,19 ± 0,14	0,92-1,46	0,06	0,811
	REM	Absolute	1,08 ± 0,14	0,80-1,35	1,15 ± 0,09	0,97-1,34	0,22	0,642
		Relative	1,18 ± 0,20	0,78-1,59	1,35 ± 0,14	1,07-1,62	0,44	0,508
Theta (4-7 Hz)	N1	Absolute	1,01 ± 0,16	0,68-1,33	1,18 ± 0,11	0,96-1,39	0,75	0,389
		Relative	0,98 ± 0,45	0,09-1,88	1,63 ± 0,31	1,02-2,24	1,40	0,240
	N2	Absolute	0,99 ± 0,16	0,67-1,31	1,11 ± 0,11	0,90-1,33	0,42	0,522
		Relative	0,91 ± 0,45	0,01-1,80	1,17 ± 0,31	0,56-1,78	0,23	0,630
	N3	Absolute	1,01 ± 0,16	0,68-1,33	1,15 ± 0,11	0,93-1,36	0,51	0,478
		Relative	0,98 ± 0,45	0,09-1,88	1,01 ± 0,31	0,39-1,63	0,00	0,963
	REM	Absolute	1,10 ± 0,16	0,78-1,43	1,17 ± 0,11	0,95-1,39	0,12	0,729
		Relative	1,23 ± 0,45	0,33-2,12	1,53 ± 0,31	0,93-2,14	0,32	0,574
Alpha (7-11 Hz)	N1	Absolute	0,85 ± 0,12	0,61-1,08	0,87 ± 0,08	0,71-1,02	0,02	0,881
		Relative	0,82 ± 0,25	0,32-1,33	1,08 ± 0,17	0,74-1,42	0,70	0,406
	N2	Absolute	0,93 ± 0,12	0,69-1,16	0,99 ± 0,08	0,84-1,15	0,22	0,644
		Relative	0,85 ± 0,25	0,35-1,35	1,02 ± 0,17	0,68-1,36	0,33	0,570
	N3	Absolute	1,13 ± 0,12	0,90-1,36	1,19 ± 0,08	1,03-1,34	0,16	0,691
		Relative	1,10 ± 0,25	0,60-1,60	1,07 ± 0,17	0,73-1,41	0,01	0,920
	REM	Absolute	0,82 ± 0,12	0,59-1,06	0,88 ± 0,08	0,72-1,04	0,18	0,675
		Relative	0,92 ± 0,25	0,42-1,43	1,12 ± 0,17	0,78-1,46	0,43	0,512

Sigma (11-14 Hz)	N1	Absolute	0,91 ± 0,10	0,71-1,11	0,98 ± 0,07	0,84-1,11	0,30	0,587
		Relative	0,89 ± 0,20	0,48-1,29	1,16 ± 0,14	0,88-1,44	1,23	0,270
	N2	Absolute	0,99 ± 0,10	0,79-1,19	1,08 ± 0,07	0,94-1,21	0,51	0,479
		Relative	0,91 ± 0,20	0,50-1,31	1,10 ± 0,14	0,83-1,38	0,64	0,426
	N3	Absolute	1,11 ± 0,10	0,90-1,31	1,17 ± 0,07	1,03-1,31	0,29	0,594
		Relative	1,08 ± 0,20	0,67-1,49	1,07 ± 0,14	0,80-1,35	0,00	0,980
	REM	Absolute	0,80 ± 0,10	0,60-1,00	0,84 ± 0,07	0,70-0,97	0,09	0,760
		Relative	0,89 ± 0,20	0,48-1,29	1,01 ± 0,14	0,73-1,28	0,25	0,616
Beta1 (14-20 Hz)	N1	Absolute	1,00 ± 0,07	0,86-1,14	1,03 ± 0,05	0,93-1,12	0,10	0,752
		Relative	0,98 ± 0,15	0,68-1,28	1,18 ± 0,10	0,97-1,38	1,19	0,278
	N2	Absolute	0,90 ± 0,07	0,75-1,04	0,95 ± 0,05	0,85-1,04	0,34	0,560
		Relative	0,82 ± 0,15	0,52-1,12	0,96 ± 0,10	0,76-1,16	0,64	0,426
	N3	Absolute	0,87 ± 0,07	0,73-1,02	0,91 ± 0,05	0,81-1,00	0,15	0,695
		Relative	0,85 ± 0,15	0,56-1,15	0,84 ± 0,10	0,63-1,04	0,01	0,928
	REM	Absolute	0,97 ± 0,07	0,82-1,11	0,96 ± 0,05	0,86-1,05	0,01	0,922
		Relative	1,07 ± 0,15	0,77-1,36	1,12 ± 0,10	0,92-1,32	0,09	0,768
Beta2 (20-35 Hz)	N1	Absolute	1,14 ± 0,05	1,04-1,25	1,15 ± 0,04	1,08-1,22	0,01	0,913
		Relative	1,11 ± 0,14	0,83-1,39	1,30 ± 0,10	1,11-1,49	1,28	0,259
	N2	Absolute	0,99 ± 0,05	0,88-1,10	1,00 ± 0,04	0,93-1,07	0,02	0,882
		Relative	0,91 ± 0,14	0,63-1,19	0,99 ± 0,10	0,80-1,18	0,21	0,647
	N3	Absolute	0,98 ± 0,05	0,87-1,09	0,97 ± 0,04	0,90-1,04	0,03	0,868
		Relative	0,96 ± 0,14	0,68-1,24	0,92 ± 0,10	0,73-1,11	0,06	0,813
	REM	Absolute	1,11 ± 0,05	1,00-1,21	1,14 ± 0,04	1,07-1,21	0,25	0,617
		Relative	1,22 ± 0,14	0,94-1,50	1,33 ± 0,10	1,14-1,52	0,46	0,496
Gamma (35-60 Hz)	N1	Absolute	1,27 ± 0,07	1,13-1,41	1,16 ± 0,05	1,07-1,25	1,63	0,203
		Relative	1,20 ± 0,09	1,02-1,38	1,24 ± 0,06	1,12-1,37	0,15	0,703
	N2	Absolute	1,08 ± 0,07	0,94-1,22	1,07 ± 0,05	0,98-1,16	0,02	0,886
		Relative	1,00 ± 0,09	0,82-1,18	1,05 ± 0,06	0,93-1,17	0,18	0,671
	N3	Absolute	1,13 ± 0,07	0,99-1,27	1,03 ± 0,05	0,94-1,12	1,42	0,235
		Relative	1,12 ± 0,09	0,94-1,30	0,98 ± 0,06	0,85-1,10	1,60	0,207
	REM	Absolute	1,07 ± 0,07	0,93-1,21	1,03 ± 0,05	0,94-1,12	0,26	0,613
		Relative	1,19 ± 0,09	1,01-1,37	1,18 ± 0,06	1,05-1,30	0,01	0,936

[a]$p < 0.05$; [b]$p \leq 0.006$.

Table S10. – Mean right centro-parietal (C4/P4) asymmetry of good sleepers and insomnia sufferers.

Frequency	Stage	Type	GS		INS		*F*	Sig.
			Mean ± SD	95% CI	Mean ± SD	95% CI		
Slow waves (0-1 Hz)	N1	Absolute	1,20 ± 0,30	0,61-1,80	1,23 ± 0,21	0,82-1,64	0,01	0,929
		Relative	1,07 ± 0,04	1,00-1,15	1,13 ± 0,03	1,08-1,19	1,39	0,239
	N2	Absolute	1,17 ± 0,30	0,58-1,76	1,10 ± 0,20	0,70-1,50	0,04	0,842
		Relative	1,01 ± 0,04	0,93-1,09	1,00 ± 0,03	0,95-1,05	0,04	0,851
	N3	Absolute	1,02 ± 0,30	0,42-1,61	0,98 ± 0,21	0,57-1,40	0,01	0,926
		Relative	0,90 ± 0,04	0,82-0,98	0,91 ± 0,03	0,86-0,96	0,03	0,868
	REM	Absolute	1,19 ± 0,30	0,59-1,78	1,79 ± 0,20	1,39-2,19	2,77	0,097
		Relative	1,08 ± 0,04	1,00-1,16	1,21 ± 0,03	1,15-1,26	7,07	**0,008**[a]
Delta (1-4 Hz)	N1	Absolute	1,16 ± 0,04	1,07-1,25	1,16 ± 0,03	1,10-1,22	0,01	0,923
		Relative	1,06 ± 0,04	0,98-1,13	1,11 ± 0,03	1,05-1,16	1,15	0,285
	N2	Absolute	1,25 ± 0,04	1,16-1,34	1,20 ± 0,03	1,14-1,26	0,79	0,375
		Relative	1,08 ± 0,04	1,01-1,16	1,16 ± 0,03	1,10-1,21	2,40	0,123
	N3	Absolute	1,24 ± 0,04	1,15-1,32	1,19 ± 0,03	1,13-1,25	0,59	0,442
		Relative	1,10 ± 0,04	1,02-1,18	1,13 ± 0,03	1,08-1,18	0,44	0,508
	REM	Absolute	1,13 ± 0,04	1,05-1,22	1,20 ± 0,03	1,14-1,26	1,44	0,232
		Relative	1,09 ± 0,04	1,02-1,17	1,05 ± 0,03	1,00-1,10	0,88	0,348
Theta (4-7 Hz)	N1	Absolute	1,03 ± 0,03	0,97-1,09	0,99 ± 0,02	0,95-1,03	1,54	0,217
		Relative	0,94 ± 0,05	0,84-1,04	0,94 ± 0,04	0,87-1,01	0,00	0,997
	N2	Absolute	1,03 ± 0,03	0,97-1,09	0,97 ± 0,02	0,93-1,01	3,23	0,076
		Relative	0,90 ± 0,05	0,80-1,00	0,96 ± 0,03	0,89-1,03	1,09	0,297
	N3	Absolute	1,05 ± 0,03	0,99-1,10	0,99 ± 0,02	0,96-1,03	2,24	0,138
		Relative	0,93 ± 0,05	0,83-1,04	0,95 ± 0,04	0,88-1,02	0,07	0,798
	REM	Absolute	1,11 ± 0,03	1,06-1,17	1,04 ± 0,02	1,00-1,08	4,50	**0,036**[a]
		Relative	1,10 ± 0,05	1,00-1,20	0,95 ± 0,03	0,88-1,02	5,49	**0,020**[a]
Alpha (7-11 Hz)	N1	Absolute	0,90 ± 0,03	0,84-0,96	0,82 ± 0,02	0,78-0,86	5,60	**0,020**[a]
		Relative	0,83 ± 0,05	0,73-0,93	0,78 ± 0,04	0,71-0,85	0,62	0,432
	N2	Absolute	0,99 ± 0,03	0,93-1,05	0,92 ± 0,02	0,88-0,96	3,45	0,066
		Relative	0,87 ± 0,05	0,76-0,97	0,92 ± 0,04	0,85-0,99	0,69	0,406
	N3	Absolute	1,13 ± 0,03	1,07-1,19	1,05 ± 0,02	1,01-1,09	5,36	**0,023**[a]
		Relative	1,01 ± 0,05	0,91-1,12	1,00 ± 0,04	0,92-1,07	0,07	0,797
	REM	Absolute	0,86 ± 0,03	0,80-0,92	0,80 ± 0,02	0,76-0,84	3,20	0,077
		Relative	0,85 ± 0,05	0,75-0,95	0,73 ± 0,04	0,66-0,80	3,50	0,062

Sigma (11-14 Hz)	N1	Absolute	0,95 ± 0,03	0,88-1,02	0,92 ± 0,02	0,88-0,97	0,45	0,504
		Relative	0,87 ± 0,04	0,78-0,96	0,88 ± 0,03	0,82-0,94	0,05	0,826
	N2	Absolute	1,05 ± 0,03	0,98-1,12	0,99 ± 0,02	0,94-1,03	2,69	0,104
		Relative	0,92 ± 0,04	0,83-1,01	0,96 ± 0,03	0,90-1,02	0,71	0,401
	N3	Absolute	1,14 ± 0,03	1,07-1,20	1,06 ± 0,02	1,02-1,11	3,24	0,075
		Relative	1,01 ± 0,04	0,92-1,10	1,01 ± 0,03	0,95-1,07	0,00	0,967
	REM	Absolute	0,84 ± 0,03	0,77-0,91	0,82 ± 0,02	0,77-0,86	0,39	0,534
		Relative	0,82 ± 0,04	0,73-0,91	0,75 ± 0,03	0,69-0,81	1,68	0,196
Beta1 (14-20 Hz)	N1	Absolute	1,03 ± 0,03	0,97-1,09	1,00 ± 0,02	0,96-1,04	0,77	0,384
		Relative	0,95 ± 0,05	0,84-1,05	0,96 ± 0,04	0,89-1,03	0,06	0,810
	N2	Absolute	0,95 ± 0,03	0,89-1,00	0,89 ± 0,02	0,85-0,92	3,05	0,084
		Relative	0,82 ± 0,05	0,72-0,92	0,89 ± 0,04	0,82-0,95	1,10	0,294
	N3	Absolute	0,93 ± 0,03	0,87-0,99	0,88 ± 0,02	0,84-0,92	2,03	0,158
		Relative	0,83 ± 0,05	0,72-0,93	0,84 ± 0,04	0,77-0,92	0,08	0,774
	REM	Absolute	1,00 ± 0,03	0,94-1,06	0,95 ± 0,02	0,91-0,99	2,05	0,156
		Relative	0,98 ± 0,05	0,88-1,08	0,89 ± 0,04	0,82-0,96	2,27	0,133
Beta2 (20-35 Hz)	N1	Absolute	1,14 ± 0,04	1,06-1,21	1,11 ± 0,03	1,06-1,16	0,29	0,591
		Relative	1,04 ± 0,06	0,93-1,16	1,07 ± 0,04	0,99-1,15	0,13	0,716
	N2	Absolute	1,04 ± 0,04	0,96-1,11	1,00 ± 0,03	0,95-1,05	0,67	0,414
		Relative	0,91 ± 0,06	0,79-1,02	0,99 ± 0,04	0,91-1,07	1,42	0,235
	N3	Absolute	1,02 ± 0,04	0,95-1,10	0,96 ± 0,03	0,91-1,02	1,62	0,205
		Relative	0,92 ± 0,06	0,80-1,04	0,92 ± 0,04	0,84-1,00	0,00	0,958
	REM	Absolute	1,15 ± 0,04	1,07-1,22	1,13 ± 0,03	1,08-1,18	0,21	0,644
		Relative	1,12 ± 0,06	1,00-1,24	1,05 ± 0,04	0,97-1,12	1,11	0,293
Gamma (35-60 Hz)	N1	Absolute	1,21 ± 0,06	1,09-1,34	1,12 ± 0,04	1,03-1,20	1,54	0,217
		Relative	1,10 ± 0,07	0,97-1,23	1,07 ± 0,05	0,98-1,16	0,15	0,703
	N2	Absolute	1,07 ± 0,06	0,94-1,19	1,07 ± 0,04	0,98-1,15	0,00	0,992
		Relative	0,94 ± 0,07	0,81-1,07	1,04 ± 0,05	0,95-1,13	1,66	0,199
	N3	Absolute	1,12 ± 0,06	0,99-1,24	1,02 ± 0,04	0,94-1,11	1,44	0,233
		Relative	1,01 ± 0,07	0,87-1,14	0,97 ± 0,05	0,88-1,06	0,20	0,652
	REM	Absolute	1,06 ± 0,06	0,93-1,19	1,06 ± 0,04	0,97-1,14	0,00	0,981
		Relative	1,03 ± 0,07	0,90-1,16	0,94 ± 0,05	0,85-1,03	1,22	0,271

[a]$p < 0.05$; [b]$p \leq 0.006$.

Table S11. – Mean mid centro-parietal (Cz/Pz) asymmetry of good sleepers and insomnia sufferers.

Frequency	Stage	Type	GS		INS		*F*	Sig.
			**Mean ± SD**	**95% CI**	**Mean ± SD**	**95% CI**		
Slow waves (0-1 Hz)	N1	Absolute	1,08 ± 0,12	0,85-1,31	1,22 ± 0,08	1,06-1,38	1,00	0,317
		Relative	0,89 ± 0,04	0,82-0,96	0,91 ± 0,03	0,86-0,96	0,23	0,634
	N2	Absolute	1,07 ± 0,12	0,84-1,29	1,08 ± 0,08	0,93-1,24	0,02	0,892
		Relative	0,88 ± 0,04	0,81-0,96	0,89 ± 0,03	0,84-0,94	0,05	0,820
	N3	Absolute	0,95 ± 0,12	0,72-1,18	0,98 ± 0,08	0,82-1,14	0,04	0,842
		Relative	0,84 ± 0,04	0,77-0,92	0,85 ± 0,03	0,80-0,90	0,01	0,913
	REM	Absolute	1,07 ± 0,12	0,84-1,30	1,06 ± 0,08	0,90-1,21	0,00	0,946
		Relative	0,87 ± 0,04	0,79-0,94	0,86 ± 0,03	0,81-0,91	0,00	0,953
Delta (1-4 Hz)	N1	Absolute	1,39 ± 0,04	1,32-1,47	1,44 ± 0,03	1,39-1,50	1,03	0,312
		Relative	1,14 ± 0,03	1,08-1,21	1,22 ± 0,02	1,18-1,27	3,81	0,053
	N2	Absolute	1,36 ± 0,04	1,28-1,44	1,41 ± 0,03	1,35-1,46	1,06	0,304
		Relative	1,17 ± 0,03	1,10-1,24	1,17 ± 0,02	1,13-1,21	0,00	0,992
	N3	Absolute	1,27 ± 0,04	1,19-1,35	1,32 ± 0,03	1,27-1,37	1,04	0,310
		Relative	1,17 ± 0,03	1,11-1,24	1,14 ± 0,02	1,10-1,19	0,50	0,481
	REM	Absolute	1,38 ± 0,04	1,31-1,46	1,40 ± 0,03	1,35-1,45	0,13	0,721
		Relative	1,12 ± 0,03	1,06-1,19	1,15 ± 0,02	1,11-1,20	0,60	0,441
Theta (4-7 Hz)	N1	Absolute	1,27 ± 0,04	1,19-1,35	1,28 ± 0,03	1,22-1,33	0,02	0,877
		Relative	1,04 ± 0,04	0,96-1,12	1,09 ± 0,03	1,04-1,15	1,23	0,269
	N2	Absolute	1,19 ± 0,04	1,11-1,27	1,18 ± 0,03	1,13-1,24	0,01	0,908
		Relative	1,03 ± 0,04	0,95-1,11	0,99 ± 0,03	0,94-1,04	0,63	0,430
	N3	Absolute	1,18 ± 0,04	1,10-1,26	1,19 ± 0,03	1,13-1,24	0,02	0,894
		Relative	1,09 ± 0,04	1,01-1,17	1,03 ± 0,03	0,98-1,09	1,39	0,240
	REM	Absolute	1,50 ± 0,04	1,42-1,58	1,43 ± 0,03	1,38-1,49	1,78	0,186
		Relative	1,22 ± 0,04	1,14-1,30	1,18 ± 0,03	1,13-1,23	0,62	0,434
Alpha (7-11 Hz)	N1	Absolute	0,96 ± 0,05	0,87-1,05	0,92 ± 0,03	0,85-0,98	0,52	0,472
		Relative	0,79 ± 0,05	0,70-0,88	0,79 ± 0,03	0,72-0,85	0,00	0,962
	N2	Absolute	1,00 ± 0,05	0,91-1,10	1,01 ± 0,03	0,95-1,07	0,00	0,955
		Relative	0,87 ± 0,05	0,78-0,97	0,84 ± 0,03	0,78-0,91	0,26	0,611
	N3	Absolute	1,24 ± 0,05	1,15-1,33	1,22 ± 0,03	1,16-1,28	0,11	0,743
		Relative	1,15 ± 0,05	1,05-1,24	1,06 ± 0,03	1,00-1,13	2,11	0,149
	REM	Absolute	0,99 ± 0,05	0,89-1,08	0,97 ± 0,03	0,91-1,04	0,07	0,792
		Relative	0,80 ± 0,05	0,71-0,89	0,80 ± 0,03	0,74-0,86	0,00	0,996

Sigma (11-14 Hz)	N1	Absolute	1,07 ± 0,05	0,97-1,17	1,06 ± 0,03	0,99-1,12	0,06	0,811
		Relative	0,88 ± 0,05	0,78-0,98	0,91 ± 0,03	0,85-0,98	0,27	0,604
	N2	Absolute	1,07 ± 0,05	0,97-1,16	1,05 ± 0,03	0,99-1,11	0,10	0,750
		Relative	0,93 ± 0,05	0,84-1,03	0,87 ± 0,03	0,81-0,94	1,04	0,310
	N3	Absolute	1,24 ± 0,05	1,15-1,34	1,22 ± 0,03	1,15-1,28	0,15	0,696
		Relative	1,16 ± 0,05	1,06-1,26	1,06 ± 0,03	0,99-1,13	2,90	0,090
	REM	Absolute	0,95 ± 0,05	0,86-1,05	0,95 ± 0,03	0,89-1,01	0,01	0,924
		Relative	0,77 ± 0,05	0,68-0,87	0,78 ± 0,03	0,72-0,85	0,02	0,881
Beta1 (14-20 Hz)	N1	Absolute	1,23 ± 0,04	1,15-1,30	1,22 ± 0,02	1,17-1,27	0,00	0,980
		Relative	1,01 ± 0,04	0,94-1,08	1,06 ± 0,03	1,01-1,11	1,48	0,224
	N2	Absolute	1,02 ± 0,04	0,94-1,09	0,98 ± 0,02	0,93-1,03	0,72	0,399
		Relative	0,88 ± 0,04	0,80-0,95	0,81 ± 0,02	0,76-0,86	1,93	0,166
	N3	Absolute	1,01 ± 0,04	0,94-1,09	1,00 ± 0,03	0,95-1,05	0,16	0,691
		Relative	0,93 ± 0,04	0,85-1,00	0,87 ± 0,03	0,82-0,92	1,62	0,204
	REM	Absolute	1,17 ± 0,04	1,09-1,24	1,14 ± 0,02	1,09-1,19	0,33	0,569
		Relative	0,95 ± 0,04	0,87-1,02	0,95 ± 0,02	0,90-0,99	0,00	0,955
Beta2 (20-35 Hz)	N1	Absolute	1,47 ± 0,06	1,34-1,60	1,50 ± 0,04	1,42-1,59	0,19	0,664
		Relative	1,21 ± 0,06	1,09-1,33	1,30 ± 0,04	1,22-1,38	1,56	0,213
	N2	Absolute	1,22 ± 0,06	1,09-1,35	1,21 ± 0,04	1,12-1,29	0,02	0,878
		Relative	1,06 ± 0,06	0,94-1,17	1,01 ± 0,04	0,93-1,09	0,46	0,498
	N3	Absolute	1,22 ± 0,06	1,09-1,34	1,18 ± 0,04	1,09-1,26	0,26	0,614
		Relative	1,12 ± 0,06	1,00-1,24	1,03 ± 0,04	0,95-1,11	1,79	0,183
	REM	Absolute	1,42 ± 0,06	1,29-1,55	1,46 ± 0,04	1,38-1,55	0,35	0,557
		Relative	1,15 ± 0,06	1,04-1,27	1,20 ± 0,04	1,12-1,28	0,49	0,487
Gamma (35-60 Hz)	N1	Absolute	1,21 ± 0,06	1,08-1,33	1,27 ± 0,04	1,19-1,36	0,75	0,389
		Relative	1,00 ± 0,07	0,87-1,13	1,10 ± 0,05	1,01-1,18	1,43	0,236
	N2	Absolute	1,21 ± 0,06	1,08-1,34	1,20 ± 0,04	1,11-1,29	0,03	0,870
		Relative	1,05 ± 0,07	0,92-1,19	1,03 ± 0,04	0,94-1,12	0,10	0,748
	N3	Absolute	1,20 ± 0,06	1,08-1,33	1,13 ± 0,04	1,04-1,22	0,92	0,339
		Relative	1,11 ± 0,07	0,98-1,25	0,99 ± 0,05	0,90-1,08	2,25	0,137
	REM	Absolute	1,28 ± 0,06	1,15-1,41	1,26 ± 0,04	1,18-1,35	0,05	0,826
		Relative	1,05 ± 0,07	0,91-1,18	1,06 ± 0,04	0,97-1,14	0,01	0,910

$^{a}p < 0.05$; $^{b}p \leq 0.006$.

Table S12. – Mean left centro-occipital (C3/O1) asymmetry of good sleepers and insomnia sufferers.

Frequency	Stage	Type	GS		INS		*F*	Sig.
			Mean ± SD	95% CI	Mean ± SD	95% CI		
Slow waves (0-1 Hz)	N1	Absolute	1,00 ± 0,09	0,83-1,17	0,95 ± 0,06	0,84-1,07	0,17	0,680
		Relative	0,89 ± 0,04	0,81-0,97	0,91 ± 0,03	0,86-0,97	0,17	0,682
	N2	Absolute	1,63 ± 0,09	1,46-1,80	1,37 ± 0,06	1,26-1,49	5,89	**0,017**[a]
		Relative	1,06 ± 0,04	0,98-1,14	1,03 ± 0,03	0,97-1,08	0,61	0,438
	N3	Absolute	1,29 ± 0,09	1,12-1,46	1,25 ± 0,06	1,13-1,36	0,18	0,671
		Relative	0,83 ± 0,04	0,75-0,90	0,84 ± 0,03	0,79-0,90	0,12	0,733
	REM	Absolute	0,95 ± 0,09	0,78-1,12	0,90 ± 0,06	0,79-1,02	0,22	0,642
		Relative	0,84 ± 0,04	0,76-0,92	0,93 ± 0,03	0,87-0,98	3,24	0,074
Delta (1-4 Hz)	N1	Absolute	1,34 ± 0,09	1,17-1,51	1,34 ± 0,06	1,22-1,46	0,00	0,978
		Relative	1,20 ± 0,03	1,14-1,25	1,27 ± 0,02	1,23-1,31	4,79	**0,030**[a]
	N2	Absolute	1,76 ± 0,09	1,59-1,93	1,64 ± 0,06	1,53-1,76	1,39	0,242
		Relative	1,18 ± 0,03	1,12-1,23	1,24 ± 0,02	1,20-1,28	3,69	0,057
	N3	Absolute	1,92 ± 0,09	1,74-2,09	1,85 ± 0,06	1,73-1,97	0,38	0,538
		Relative	1,26 ± 0,03	1,21-1,31	1,26 ± 0,02	1,23-1,30	0,01	0,916
	REM	Absolute	1,30 ± 0,09	1,13-1,47	1,13 ± 0,06	1,01-1,24	2,64	0,108
		Relative	1,16 ± 0,03	1,11-1,22	1,17 ± 0,02	1,14-1,21	0,10	0,751
Theta (4-7 Hz)	N1	Absolute	1,01 ± 0,07	0,87-1,15	0,94 ± 0,05	0,84-1,04	0,66	0,419
		Relative	0,91 ± 0,03	0,84-0,98	0,89 ± 0,02	0,84-0,93	0,28	0,597
	N2	Absolute	0,95 ± 0,07	0,81-1,09	0,87 ± 0,05	0,77-0,96	0,96	0,329
		Relative	0,63 ± 0,03	0,56-0,70	0,65 ± 0,02	0,61-0,70	0,28	0,595
	N3	Absolute	1,06 ± 0,07	0,92-1,20	0,97 ± 0,05	0,88-1,07	1,08	0,303
		Relative	0,70 ± 0,03	0,63-0,77	0,67 ± 0,02	0,62-0,72	0,54	0,463
	REM	Absolute	1,35 ± 0,07	1,21-1,49	1,03 ± 0,05	0,93-1,12	**14,03**	**0,000**[b]
		Relative	1,20 ± 0,03	1,13-1,27	1,07 ± 0,02	1,02-1,12	**9,78**	**0,002**[b]
Alpha (7-11 Hz)	N1	Absolute	0,89 ± 0,07	0,75-1,04	0,78 ± 0,05	0,69-0,88	1,58	0,212
		Relative	0,81 ± 0,04	0,73-0,89	0,76 ± 0,03	0,70-0,81	1,18	0,279
	N2	Absolute	1,12 ± 0,07	0,98-1,27	0,96 ± 0,05	0,87-1,06	3,52	0,064
		Relative	0,76 ± 0,04	0,68-0,84	0,73 ± 0,03	0,68-0,79	0,41	0,523
	N3	Absolute	1,43 ± 0,07	1,28-1,57	1,22 ± 0,05	1,12-1,32	5,69	**0,019**[a]
		Relative	0,95 ± 0,04	0,87-1,03	0,84 ± 0,03	0,79-0,89	5,30	**0,022**[a]
	REM	Absolute	0,88 ± 0,07	0,74-1,03	0,73 ± 0,05	0,64-0,83	3,00	0,087
		Relative	0,79 ± 0,04	0,72-0,87	0,77 ± 0,03	0,71-0,82	0,36	0,551

Sigma (11-14 Hz)	N1	Absolute	1,04 ± 0,10	0,85-1,23	0,97 ± 0,07	0,84-1,10	0,32	0,570
		Relative	0,94 ± 0,05	0,84-1,05	0,94 ± 0,04	0,86-1,01	0,01	0,932
	N2	Absolute	1,69 ± 0,10	1,50-1,89	1,43 ± 0,06	1,31-1,56	4,93	**0,029**[a]
		Relative	1,12 ± 0,05	1,02-1,22	1,10 ± 0,04	1,03-1,17	0,10	0,748
	N3	Absolute	1,80 ± 0,10	1,61-1,99	1,55 ± 0,07	1,42-1,68	4,60	**0,035**[a]
		Relative	1,14 ± 0,05	1,04-1,25	1,10 ± 0,04	1,03-1,18	0,37	0,542
	REM	Absolute	0,85 ± 0,10	0,66-1,04	0,73 ± 0,06	0,60-0,86	1,14	0,288
		Relative	0,76 ± 0,05	0,66-0,87	0,77 ± 0,04	0,70-0,84	0,02	0,878
Beta1 (14-20 Hz)	N1	Absolute	1,27 ± 0,08	1,12-1,42	1,16 ± 0,05	1,05-1,26	1,55	0,217
		Relative	1,15 ± 0,05	1,05-1,25	1,13 ± 0,03	1,06-1,20	0,08	0,782
	N2	Absolute	1,44 ± 0,08	1,29-1,59	1,25 ± 0,05	1,15-1,35	4,53	**0,036**[a]
		Relative	0,97 ± 0,05	0,88-1,07	0,97 ± 0,03	0,91-1,04	0,00	0,988
	N3	Absolute	1,28 ± 0,08	1,13-1,43	1,19 ± 0,05	1,09-1,29	1,07	0,305
		Relative	0,85 ± 0,05	0,75-0,95	0,84 ± 0,03	0,78-0,91	0,01	0,909
	REM	Absolute	1,14 ± 0,08	0,99-1,29	0,98 ± 0,05	0,88-1,08	3,16	0,080
		Relative	1,01 ± 0,05	0,91-1,11	1,06 ± 0,03	0,99-1,12	0,59	0,443
Beta2 (20-35 Hz)	N1	Absolute	1,32 ± 0,09	1,15-1,49	1,25 ± 0,06	1,13-1,36	0,47	0,493
		Relative	1,20 ± 0,07	1,06-1,34	1,22 ± 0,05	1,13-1,32	0,09	0,762
	N2	Absolute	1,09 ± 0,09	0,92-1,26	0,99 ± 0,06	0,88-1,11	0,89	0,347
		Relative	0,75 ± 0,07	0,61-0,89	0,77 ± 0,05	0,68-0,87	0,08	0,776
	N3	Absolute	1,03 ± 0,09	0,86-1,20	0,97 ± 0,06	0,85-1,08	0,39	0,536
		Relative	0,70 ± 0,07	0,57-0,84	0,69 ± 0,05	0,59-0,78	0,04	0,835
	REM	Absolute	1,42 ± 0,09	1,25-1,59	1,26 ± 0,06	1,15-1,38	2,33	0,130
		Relative	1,24 ± 0,07	1,10-1,37	1,38 ± 0,05	1,29-1,47	2,92	0,090
Gamma (35-60 Hz)	N1	Absolute	1,29 ± 0,09	1,12-1,46	1,14 ± 0,06	1,02-1,25	2,32	0,130
		Relative	1,18 ± 0,08	1,02-1,34	1,11 ± 0,06	1,00-1,22	0,46	0,497
	N2	Absolute	1,07 ± 0,09	0,90-1,24	1,01 ± 0,06	0,90-1,12	0,38	0,541
		Relative	0,75 ± 0,08	0,59-0,92	0,80 ± 0,06	0,69-0,91	0,20	0,654
	N3	Absolute	1,11 ± 0,09	0,95-1,28	0,95 ± 0,06	0,83-1,06	2,57	0,111
		Relative	0,80 ± 0,08	0,64-0,96	0,67 ± 0,06	0,56-0,78	1,70	0,194
	REM	Absolute	1,13 ± 0,09	0,96-1,30	0,98 ± 0,06	0,87-1,09	2,10	0,149
		Relative	1,03 ± 0,08	0,87-1,19	1,07 ± 0,06	0,96-1,18	0,16	0,690

[a]$p < 0.05$; [b]$p \leq 0.006$.

Table S13. – Mean right centro-occipital (C4/O2) asymmetry of good sleepers and insomnia sufferers.

Frequency	Stage	Type	GS		INS		F	Sig.
			Mean ± SD	95% CI	Mean ± SD	95% CI		
Slow waves (0-1 Hz)	N1	Absolute	1,31 ± 0,37	0,57-2,04	1,32 ± 0,26	0,81-1,82	0,00	0,984
		Relative	1,01 ± 0,05	0,92-1,11	1,08 ± 0,03	1,01-1,15	1,22	0,270
	N2	Absolute	1,76 ± 0,37	1,03-2,49	1,45 ± 0,25	0,96-1,95	0,47	0,494
		Relative	1,05 ± 0,05	0,96-1,15	1,02 ± 0,03	0,96-1,09	0,29	0,594
	N3	Absolute	1,50 ± 0,37	0,77-2,23	1,37 ± 0,26	0,86-1,88	0,08	0,778
		Relative	0,85 ± 0,05	0,75-0,95	0,87 ± 0,03	0,80-0,94	0,12	0,733
	REM	Absolute	1,47 ± 0,37	0,74-2,20	1,97 ± 0,25	1,48-2,47	1,23	0,267
		Relative	1,07 ± 0,05	0,97-1,17	1,20 ± 0,03	1,13-1,27	4,74	**0,031**[a]
Delta (1-4 Hz)	N1	Absolute	1,38 ± 0,10	1,18-1,58	1,40 ± 0,07	1,26-1,53	0,01	0,921
		Relative	1,23 ± 0,05	1,14-1,32	1,21 ± 0,03	1,15-1,27	0,15	0,702
	N2	Absolute	1,91 ± 0,10	1,71-2,11	1,73 ± 0,07	1,60-1,86	2,12	0,148
		Relative	1,18 ± 0,05	1,09-1,27	1,23 ± 0,03	1,17-1,29	0,89	0,346
	N3	Absolute	2,16 ± 0,10	1,96-2,36	2,00 ± 0,07	1,86-2,14	1,78	0,185
		Relative	1,26 ± 0,05	1,17-1,35	1,25 ± 0,03	1,19-1,31	0,02	0,885
	REM	Absolute	1,46 ± 0,10	1,26-1,66	1,36 ± 0,07	1,22-1,49	0,75	0,389
		Relative	1,09 ± 0,05	1,00-1,18	1,11 ± 0,03	1,05-1,18	0,15	0,701
Theta (4-7 Hz)	N1	Absolute	1,05 ± 0,07	0,91-1,19	0,99 ± 0,05	0,89-1,09	0,48	0,490
		Relative	1,49 ± 0,19	1,11-1,87	0,86 ± 0,13	0,60-1,12	7,43	**0,007**[b]
	N2	Absolute	1,07 ± 0,07	0,92-1,21	0,94 ± 0,05	0,84-1,03	2,18	0,144
		Relative	0,65 ± 0,19	0,27-1,03	0,67 ± 0,13	0,42-0,93	0,01	0,913
	N3	Absolute	1,19 ± 0,07	1,05-1,34	1,06 ± 0,05	0,96-1,16	2,38	0,127
		Relative	0,69 ± 0,19	0,31-1,07	0,67 ± 0,13	0,41-0,93	0,01	0,924
	REM	Absolute	1,40 ± 0,07	1,26-1,55	1,09 ± 0,05	0,99-1,18	**13,36**	**0,000**[b]
		Relative	1,04 ± 0,19	0,66-1,42	0,95 ± 0,13	0,69-1,20	0,15	0,698
Alpha (7-11 Hz)	N1	Absolute	1,01 ± 0,07	0,87-1,16	0,91 ± 0,05	0,81-1,01	1,28	0,260
		Relative	1,48 ± 0,19	1,11-1,85	0,80 ± 0,13	0,55-1,06	**8,83**	**0,003**[b]
	N2	Absolute	1,30 ± 0,07	1,16-1,45	1,10 ± 0,05	1,00-1,20	5,24	**0,024**[a]
		Relative	0,81 ± 0,19	0,44-1,18	0,79 ± 0,13	0,54-1,04	0,01	0,924
	N3	Absolute	1,54 ± 0,07	1,40-1,69	1,27 ± 0,05	1,17-1,37	**9,48**	**0,003**[b]
		Relative	0,89 ± 0,19	0,52-1,26	0,81 ± 0,13	0,55-1,07	0,14	0,705
	REM	Absolute	0,97 ± 0,07	0,83-1,12	0,82 ± 0,05	0,73-0,92	2,85	0,095
		Relative	0,74 ± 0,19	0,37-1,11	0,71 ± 0,13	0,46-0,96	0,01	0,915

Sigma (11-14 Hz)	N1	Absolute	1,17 ± 0,09	0,99-1,36	1,10 ± 0,06	0,97-1,22	0,49	0,485
		Relative	1,59 ± 0,19	1,22-1,95	0,97 ± 0,13	0,72-1,22	**7,55**	**0,006**[b]
	N2	Absolute	1,83 ± 0,09	1,65-2,01	1,55 ± 0,06	1,43-1,67	6,40	**0,013**[a]
		Relative	1,13 ± 0,19	0,76-1,49	1,12 ± 0,13	0,87-1,36	0,00	0,962
	N3	Absolute	1,84 ± 0,09	1,65-2,02	1,57 ± 0,06	1,45-1,69	5,77	**0,018**[a]
		Relative	1,05 ± 0,19	0,68-1,41	1,01 ± 0,13	0,75-1,26	0,03	0,862
	REM	Absolute	0,97 ± 0,09	0,78-1,15	0,86 ± 0,06	0,74-0,99	0,86	0,357
		Relative	0,73 ± 0,19	0,37-1,09	0,75 ± 0,13	0,51-1,00	0,01	0,914
Beta1 (14-20 Hz)	N1	Absolute	1,36 ± 0,07	1,22-1,51	1,27 ± 0,05	1,17-1,37	1,15	0,287
		Relative	2,03 ± 0,27	1,51-2,56	1,14 ± 0,18	0,78-1,51	**7,55**	**0,006**[b]
	N2	Absolute	1,53 ± 0,07	1,38-1,67	1,34 ± 0,05	1,24-1,44	4,41	**0,039**[a]
		Relative	0,95 ± 0,27	0,43-1,47	0,98 ± 0,18	0,62-1,33	0,01	0,930
	N3	Absolute	1,36 ± 0,07	1,22-1,51	1,25 ± 0,05	1,15-1,35	1,67	0,200
		Relative	0,80 ± 0,27	0,27-1,32	0,82 ± 0,19	0,45-1,18	0,00	0,955
	REM	Absolute	1,25 ± 0,07	1,10-1,40	1,12 ± 0,05	1,02-1,21	2,32	0,132
		Relative	0,94 ± 0,27	0,41-1,46	1,01 ± 0,18	0,65-1,36	0,05	0,831
Beta2 (20-35 Hz)	N1	Absolute	1,42 ± 0,10	1,23-1,61	1,35 ± 0,06	1,22-1,48	0,37	0,547
		Relative	1,83 ± 0,20	1,44-2,23	1,23 ± 0,14	0,96-1,50	6,15	**0,014**[a]
	N2	Absolute	1,24 ± 0,10	1,05-1,43	1,12 ± 0,06	0,99-1,25	1,15	0,287
		Relative	0,79 ± 0,20	0,40-1,18	0,82 ± 0,14	0,55-1,08	0,01	0,919
	N3	Absolute	1,17 ± 0,10	0,98-1,36	1,06 ± 0,06	0,94-1,19	0,89	0,347
		Relative	0,70 ± 0,20	0,31-1,09	0,69 ± 0,14	0,42-0,97	0,00	0,980
	REM	Absolute	1,60 ± 0,10	1,41-1,79	1,41 ± 0,06	1,28-1,54	2,72	0,103
		Relative	1,17 ± 0,20	0,78-1,56	1,27 ± 0,14	1,00-1,53	0,16	0,694
Gamma (35-60 Hz)	N1	Absolute	1,42 ± 0,11	1,21-1,64	1,25 ± 0,07	1,10-1,39	1,79	0,183
		Relative	1,69 ± 0,17	1,36-2,02	1,13 ± 0,11	0,91-1,36	7,46	**0,007**[a]
	N2	Absolute	1,20 ± 0,11	0,99-1,42	1,18 ± 0,07	1,03-1,32	0,04	0,834
		Relative	0,78 ± 0,17	0,45-1,11	0,85 ± 0,11	0,63-1,07	0,12	0,724
	N3	Absolute	1,26 ± 0,11	1,04-1,47	1,13 ± 0,07	0,98-1,28	0,94	0,335
		Relative	0,77 ± 0,17	0,44-1,10	0,73 ± 0,12	0,50-0,96	0,05	0,828
	REM	Absolute	1,28 ± 0,11	1,06-1,49	1,24 ± 0,07	1,09-1,38	0,09	0,765
		Relative	0,98 ± 0,17	0,65-1,31	1,07 ± 0,11	0,85-1,29	0,22	0,636

[a]$p < 0.05$; [b]$p \leq 0.006$.

Table S14. – Mean left parieto-occipital (P3/O1) asymmetry of good sleepers and insomnia sufferers.

Frequency	Stage	Type	GS		INS		F	Sig.
			Mean ± SD	95% CI	Mean ± SD	95% CI		
Slow waves (0-1 Hz)	N1	Absolute	1,03 ± 0,43	0,17-1,88	1,65 ± 0,30	1,07-2,24	1,44	0,232
		Relative	0,95 ± 0,08	0,79-1,12	1,12 ± 0,06	1,01-1,23	2,79	0,097
	N2	Absolute	1,47 ± 0,43	0,62-2,32	1,66 ± 0,29	1,08-2,24	0,13	0,715
		Relative	1,07 ± 0,08	0,91-1,24	1,16 ± 0,06	1,05-1,27	0,78	0,378
	N3	Absolute	1,45 ± 0,43	0,60-2,31	1,40 ± 0,30	0,81-1,99	0,01	0,922
		Relative	0,95 ± 0,08	0,79-1,11	0,96 ± 0,06	0,85-1,07	0,01	0,924
	REM	Absolute	1,40 ± 0,43	0,55-2,26	1,34 ± 0,29	0,76-1,91	0,02	0,897
		Relative	1,00 ± 0,08	0,83-1,16	1,08 ± 0,06	0,97-1,19	0,64	0,424
Delta (1-4 Hz)	N1	Absolute	1,18 ± 0,06	1,05-1,30	1,12 ± 0,04	1,04-1,21	0,51	0,476
		Relative	1,09 ± 0,03	1,03-1,14	1,06 ± 0,02	1,02-1,10	0,62	0,432
	N2	Absolute	1,47 ± 0,06	1,34-1,59	1,34 ± 0,04	1,26-1,43	2,55	0,114
		Relative	1,08 ± 0,03	1,02-1,14	1,07 ± 0,02	1,03-1,11	0,04	0,833
	N3	Absolute	1,65 ± 0,06	1,52-1,77	1,54 ± 0,04	1,45-1,63	1,89	0,172
		Relative	1,12 ± 0,03	1,07-1,18	1,12 ± 0,02	1,08-1,16	0,01	0,921
	REM	Absolute	1,20 ± 0,06	1,07-1,33	1,03 ± 0,04	0,95-1,12	4,88	**0,030**[a]
		Relative	1,03 ± 0,03	0,97-1,09	1,01 ± 0,02	0,97-1,05	0,25	0,617
Theta (4-7 Hz)	N1	Absolute	0,99 ± 0,06	0,88-1,10	0,91 ± 0,04	0,83-0,98	1,49	0,226
		Relative	0,92 ± 0,03	0,86-0,99	0,86 ± 0,02	0,82-0,90	2,51	0,116
	N2	Absolute	0,95 ± 0,06	0,84-1,06	0,88 ± 0,04	0,80-0,95	1,17	0,283
		Relative	0,70 ± 0,03	0,63-0,76	0,70 ± 0,02	0,66-0,74	0,01	0,927
	N3	Absolute	1,05 ± 0,06	0,94-1,16	0,95 ± 0,04	0,88-1,03	1,92	0,170
		Relative	0,72 ± 0,03	0,66-0,78	0,70 ± 0,02	0,66-0,75	0,18	0,670
	REM	Absolute	1,21 ± 0,06	1,09-1,32	0,97 ± 0,04	0,90-1,05	**12,17**	**0,001**[b]
		Relative	1,03 ± 0,03	0,97-1,10	0,97 ± 0,02	0,92-1,01	3,23	0,075
Alpha (7-11 Hz)	N1	Absolute	1,05 ± 0,06	0,93-1,17	0,99 ± 0,04	0,91-1,08	0,61	0,436
		Relative	0,98 ± 0,04	0,90-1,06	0,95 ± 0,03	0,89-1,00	0,46	0,497
	N2	Absolute	1,21 ± 0,06	1,09-1,34	1,07 ± 0,04	0,99-1,15	3,85	0,053
		Relative	0,89 ± 0,04	0,82-0,97	0,86 ± 0,03	0,81-0,91	0,57	0,450
	N3	Absolute	1,25 ± 0,06	1,13-1,38	1,10 ± 0,04	1,02-1,19	4,09	**0,046**[a]
		Relative	0,86 ± 0,04	0,78-0,94	0,81 ± 0,03	0,76-0,86	0,92	0,341
	REM	Absolute	1,07 ± 0,06	0,95-1,20	0,92 ± 0,04	0,84-1,00	4,39	**0,039**[a]
		Relative	0,93 ± 0,04	0,85-1,01	0,92 ± 0,03	0,86-0,97	0,09	0,767

Sigma (11-14 Hz)	N1	Absolute	1,14 ± 0,07	1,00-1,27	1,04 ± 0,05	0,95-1,14	1,25	0,267
		Relative	1,06 ± 0,04	0,97-1,15	1,00 ± 0,03	0,94-1,06	1,25	0,265
	N2	Absolute	1,70 ± 0,07	1,56-1,83	1,47 ± 0,05	1,38-1,56	7,38	**0,008**[a]
		Relative	1,23 ± 0,04	1,14-1,32	1,19 ± 0,03	1,13-1,25	0,63	0,428
	N3	Absolute	1,58 ± 0,07	1,45-1,72	1,40 ± 0,05	1,31-1,49	4,75	**0,032**[a]
		Relative	1,07 ± 0,04	0,98-1,15	1,07 ± 0,03	1,01-1,13	0,00	0,962
	REM	Absolute	1,05 ± 0,07	0,92-1,19	0,92 ± 0,05	0,83-1,01	2,71	0,103
		Relative	0,91 ± 0,04	0,82-1,00	0,93 ± 0,03	0,87-0,99	0,08	0,782
Beta1 (14-20 Hz)	N1	Absolute	1,26 ± 0,07	1,12-1,39	1,15 ± 0,05	1,06-1,25	1,56	0,216
		Relative	1,17 ± 0,05	1,07-1,27	1,11 ± 0,03	1,04-1,18	0,88	0,351
	N2	Absolute	1,62 ± 0,07	1,49-1,76	1,44 ± 0,05	1,35-1,54	4,62	**0,035**[a]
		Relative	1,20 ± 0,05	1,10-1,30	1,17 ± 0,03	1,11-1,24	0,18	0,669
	N3	Absolute	1,47 ± 0,07	1,33-1,61	1,37 ± 0,05	1,27-1,46	1,52	0,221
		Relative	1,01 ± 0,05	0,91-1,11	1,04 ± 0,03	0,97-1,10	0,23	0,633
	REM	Absolute	1,16 ± 0,07	1,02-1,30	1,04 ± 0,05	0,95-1,13	1,95	0,167
		Relative	1,00 ± 0,05	0,90-1,10	1,06 ± 0,03	0,99-1,13	1,09	0,298
Beta2 (20-35 Hz)	N1	Absolute	1,14 ± 0,05	1,05-1,23	1,08 ± 0,03	1,01-1,14	1,35	0,248
		Relative	1,07 ± 0,04	0,98-1,16	1,05 ± 0,03	0,99-1,11	0,21	0,648
	N2	Absolute	1,09 ± 0,05	1,00-1,19	1,00 ± 0,03	0,94-1,06	2,69	0,105
		Relative	0,82 ± 0,04	0,73-0,91	0,81 ± 0,03	0,76-0,87	0,00	0,952
	N3	Absolute	1,04 ± 0,05	0,95-1,13	0,99 ± 0,03	0,93-1,06	0,75	0,389
		Relative	0,72 ± 0,04	0,64-0,81	0,77 ± 0,03	0,71-0,83	0,65	0,423
	REM	Absolute	1,23 ± 0,05	1,14-1,32	1,10 ± 0,03	1,04-1,16	5,34	**0,024**[a]
		Relative	1,06 ± 0,04	0,97-1,14	1,14 ± 0,03	1,08-1,19	2,17	0,143
Gamma (35-60 Hz)	N1	Absolute	0,98 ± 0,03	0,92-1,04	0,95 ± 0,02	0,91-1,00	0,49	0,487
		Relative	0,94 ± 0,04	0,86-1,01	0,93 ± 0,03	0,87-0,98	0,07	0,787
	N2	Absolute	1,00 ± 0,03	0,93-1,06	0,94 ± 0,02	0,90-0,98	2,06	0,155
		Relative	0,75 ± 0,04	0,68-0,83	0,77 ± 0,03	0,72-0,82	0,10	0,754
	N3	Absolute	0,99 ± 0,03	0,93-1,06	0,93 ± 0,02	0,88-0,97	3,00	0,087
		Relative	0,70 ± 0,04	0,63-0,78	0,72 ± 0,03	0,66-0,77	0,07	0,789
	REM	Absolute	1,05 ± 0,03	0,99-1,11	0,96 ± 0,02	0,91-1,00	5,92	**0,017**[a]
		Relative	0,92 ± 0,04	0,84-0,99	0,98 ± 0,03	0,93-1,03	1,62	0,205

[a] $p < 0.05$; [b] $p \leq 0.006$.

Table S15. – Mean right parieto-occipital (P4/O2) asymmetry of good sleepers and insomnia sufferers.

Frequency	Stage	Type	GS		INS		F	Sig.
			Mean ± SD	95% CI	Mean ± SD	95% CI		
Slow waves (0-1 Hz)	N1	Absolute	1,07 ± 0,35	0,38-1,76	1,06 ± 0,24	0,59-1,53	0,00	0,981
		Relative	0,94 ± 0,05	0,85-1,03	0,96 ± 0,03	0,89-1,02	0,12	0,730
	N2	Absolute	1,47 ± 0,35	0,78-2,16	1,87 ± 0,24	1,41-2,34	0,90	0,342
		Relative	1,05 ± 0,05	0,95-1,14	1,07 ± 0,03	1,00-1,13	0,14	0,710
	N3	Absolute	1,45 ± 0,35	0,76-2,14	1,53 ± 0,24	1,05-2,01	0,03	0,857
		Relative	0,94 ± 0,05	0,85-1,03	0,97 ± 0,03	0,90-1,03	0,20	0,652
	REM	Absolute	1,44 ± 0,35	0,75-2,12	1,06 ± 0,24	0,60-1,53	0,78	0,379
		Relative	1,02 ± 0,05	0,93-1,11	0,99 ± 0,03	0,93-1,06	0,26	0,614
Delta (1-4 Hz)	N1	Absolute	1,19 ± 0,05	1,09-1,29	1,19 ± 0,04	1,12-1,26	0,00	0,968
		Relative	1,16 ± 0,04	1,08-1,24	1,09 ± 0,03	1,04-1,15	1,71	0,192
	N2	Absolute	1,51 ± 0,05	1,40-1,61	1,43 ± 0,04	1,36-1,50	1,46	0,230
		Relative	1,09 ± 0,04	1,01-1,17	1,10 ± 0,03	1,05-1,16	0,12	0,730
	N3	Absolute	1,74 ± 0,05	1,63-1,84	1,66 ± 0,04	1,59-1,73	1,26	0,263
		Relative	1,15 ± 0,04	1,06-1,23	1,13 ± 0,03	1,07-1,18	0,17	0,681
	REM	Absolute	1,29 ± 0,05	1,19-1,39	1,11 ± 0,04	1,04-1,18	**7,80**	**0,006**[b]
		Relative	1,02 ± 0,04	0,94-1,10	1,06 ± 0,03	1,01-1,12	0,76	0,384
Theta (4-7 Hz)	N1	Absolute	1,01 ± 0,05	0,91-1,10	0,99 ± 0,03	0,92-1,05	0,12	0,731
		Relative	1,42 ± 0,16	1,12-1,73	0,91 ± 0,11	0,70-1,12	7,38	**0,007**[a]
	N2	Absolute	1,02 ± 0,05	0,92-1,11	0,96 ± 0,03	0,90-1,03	0,88	0,350
		Relative	0,72 ± 0,16	0,42-1,03	0,74 ± 0,11	0,54-0,95	0,01	0,914
	N3	Absolute	1,13 ± 0,05	1,03-1,23	1,06 ± 0,03	0,99-1,12	1,43	0,236
		Relative	0,74 ± 0,16	0,43-1,05	0,71 ± 0,11	0,50-0,93	0,02	0,893
	REM	Absolute	1,23 ± 0,05	1,14-1,33	1,02 ± 0,03	0,96-1,09	**12,41**	**0,001**[b]
		Relative	0,99 ± 0,16	0,69-1,30	0,99 ± 0,11	0,78-1,20	0,00	0,991
Alpha (7-11 Hz)	N1	Absolute	1,12 ± 0,06	1,01-1,24	1,12 ± 0,04	1,04-1,20	0,01	0,938
		Relative	1,61 ± 0,17	1,26-1,95	1,03 ± 0,12	0,79-1,27	7,42	**0,007**[a]
	N2	Absolute	1,31 ± 0,06	1,19-1,43	1,18 ± 0,04	1,10-1,26	2,98	0,088
		Relative	0,94 ± 0,17	0,59-1,28	0,92 ± 0,12	0,69-1,15	0,01	0,927
	N3	Absolute	1,33 ± 0,06	1,21-1,45	1,19 ± 0,04	1,11-1,27	3,55	0,063
		Relative	0,87 ± 0,17	0,53-1,21	0,82 ± 0,12	0,58-1,06	0,06	0,810
	REM	Absolute	1,14 ± 0,06	1,02-1,25	1,03 ± 0,04	0,95-1,11	2,22	0,140
		Relative	0,92 ± 0,17	0,58-1,26	0,98 ± 0,12	0,75-1,21	0,09	0,770

Sigma (11-14 Hz)	N1	Absolute	1,23 ± 0,06	1,10-1,36	1,17 ± 0,04	1,08-1,26	0,61	0,436
		Relative	1,73 ± 0,18	1,37-2,09	1,09 ± 0,13	0,84-1,34	**8,26**	**0,004[b]**
	N2	Absolute	1,71 ± 0,06	1,58-1,84	1,56 ± 0,04	1,48-1,65	3,67	0,059
		Relative	1,22 ± 0,18	0,86-1,58	1,21 ± 0,12	0,97-1,45	0,00	0,949
	N3	Absolute	1,56 ± 0,06	1,44-1,69	1,47 ± 0,04	1,38-1,56	1,48	0,227
		Relative	1,03 ± 0,18	0,67-1,39	1,01 ± 0,13	0,75-1,26	0,01	0,931
	REM	Absolute	1,15 ± 0,06	1,02-1,28	1,05 ± 0,04	0,96-1,13	1,65	0,203
		Relative	0,92 ± 0,18	0,56-1,28	1,00 ± 0,12	0,76-1,25	0,14	0,710
Beta1 (14-20 Hz)	N1	Absolute	1,31 ± 0,06	1,19-1,43	1,26 ± 0,04	1,18-1,34	0,50	0,481
		Relative	2,05 ± 0,25	1,55-2,55	1,19 ± 0,17	0,84-1,53	**7,86**	**0,005[b]**
	N2	Absolute	1,61 ± 0,06	1,49-1,73	1,52 ± 0,04	1,44-1,60	1,62	0,206
		Relative	1,17 ± 0,25	0,67-1,66	1,19 ± 0,17	0,85-1,52	0,00	0,947
	N3	Absolute	1,46 ± 0,06	1,34-1,58	1,42 ± 0,04	1,34-1,50	0,31	0,577
		Relative	0,97 ± 0,25	0,47-1,47	0,98 ± 0,18	0,63-1,33	0,00	0,976
	REM	Absolute	1,23 ± 0,06	1,11-1,35	1,17 ± 0,04	1,09-1,25	0,86	0,355
		Relative	0,99 ± 0,25	0,49-1,49	1,13 ± 0,17	0,79-1,47	0,20	0,656
Beta2 (20-35 Hz)	N1	Absolute	1,23 ± 0,05	1,14-1,33	1,19 ± 0,03	1,13-1,25	0,53	0,470
		Relative	1,81 ± 0,20	1,41-2,21	1,12 ± 0,14	0,85-1,40	**7,74**	**0,006[b]**
	N2	Absolute	1,19 ± 0,05	1,09-1,28	1,11 ± 0,03	1,05-1,18	1,70	0,196
		Relative	0,86 ± 0,20	0,46-1,26	0,87 ± 0,14	0,60-1,14	0,00	0,977
	N3	Absolute	1,13 ± 0,05	1,04-1,23	1,10 ± 0,03	1,03-1,16	0,36	0,550
		Relative	0,75 ± 0,20	0,35-1,15	0,76 ± 0,14	0,48-1,04	0,00	0,985
	REM	Absolute	1,32 ± 0,05	1,23-1,42	1,23 ± 0,03	1,16-1,29	2,77	0,100
		Relative	1,06 ± 0,20	0,66-1,46	1,20 ± 0,14	0,93-1,47	0,32	0,572
Gamma (35-60 Hz)	N1	Absolute	1,11 ± 0,05	1,01-1,21	1,09 ± 0,03	1,03-1,16	0,09	0,761
		Relative	1,67 ± 0,19	1,29-2,05	1,04 ± 0,13	0,78-1,30	7,29	**0,007[a]**
	N2	Absolute	1,12 ± 0,05	1,03-1,22	1,10 ± 0,03	1,04-1,17	0,15	0,696
		Relative	0,82 ± 0,19	0,44-1,20	0,86 ± 0,13	0,61-1,12	0,03	0,855
	N3	Absolute	1,11 ± 0,05	1,01-1,21	1,13 ± 0,03	1,07-1,20	0,17	0,679
		Relative	0,74 ± 0,19	0,37-1,12	0,79 ± 0,13	0,52-1,05	0,03	0,858
	REM	Absolute	1,20 ± 0,05	1,10-1,30	1,17 ± 0,03	1,10-1,23	0,39	0,533
		Relative	0,98 ± 0,19	0,60-1,36	1,13 ± 0,13	0,88-1,39	0,44	0,505

[a]$p < 0.05$; [b]$p \leq 0.006$.
